# Visual attention: Defining the origin of the current terms of engagement

**DOI:** 10.3758/s13414-026-03354-0

**Published:** 2026-10-26

**Authors:** Geoffrey F. Woodman

**Affiliations:** Department of Psychology, Vanderbilt Vision Research Center, Vanderbilt University, PMB 407817, 2301 Vanderbilt Place, Nashville, TN 37240-7817 USA

**Keywords:** Divided attention, Visual search, Spatial cueing, Attentional control, Attentional capture, Capacity limits

## Abstract

Our environment bombards the mind with far more visual information than can be processed. Because of this overload of information, our minds focus on only a subset of the visual inputs that land on our retinas. The visual attention mechanisms of the brain select which inputs we process to the level of awareness, store in memory, and act upon. Here I provide an overview of the topics, tasks, and methods that researchers have developed to study visual attention and conclude by discussing continuing controversies. Finally, weaknesses in our understanding of the brain’s limited capacity ability to process visual information are identified as potential targets for future study.

## Introduction

*“Everyone knows what attention is. It is the taking possession by the mind in clear and vivid form, of one out of what seem several simultaneously possible objects or trains of thought.”*

- William James (1890)

This classic definition of attention is both misleading and accurate. The misleading aspect of this statement is from the first sentence; that everyone already knows what attention is. The appeal to our intuition results in psychologists assuming that the scientific definition of attention maps onto their own definition of the term (Anderson et al., 2021; Hommel et al., 2019).

Given the appeal to intuition, readers of James’s definition might assume that attention researchers study how people maintain alertness (i.e., a heightened state of arousal), with a sufficient amount of arousal needed to make us consciously aware of information from our environment (Coull, 1998; Pribram & McGuiness, 1975). For example, when our mind wanders at a lecture and we miss a critical slide or definition of a term, we informally state that we wish we had *paid attention* during that portion of the lecture. A number of scientists seek to understand how mind wandering comes about in the brain (e.g., Mooneyham & Schooler, 2013). However, it should be noted that this definition of attention, as analogous to awareness, is typically only used in clinical science and sports psychology (Hill et al., 2019; Pagoto et al., 2013). Although the term is used differently in these literatures, these are productive lines of work that provide translational opportunities for our knowledge acquired about the relationship between attention, awareness, and memory storage (Luck et al., 2019; Nuechterlein et al., 2009; Ramsay et al., 2021; Reinhart et al., 2019; Woodman & Luck, 2003a). As I will focus on now, most cognitive scientists who study attention in their laboratories deal with the ideas communicated in the second sentence of James’ definition; it is the taking possession by the mind in clear and vivid form, of one out of what seem several simultaneously possible objects or trains of thought.

Visual attention allows us to focus processing on the relevant stuff and ignore the irrelevant stuff in our visual field (Broadbent, 1957; Chun & Wolfe, 2001; Desimone & Duncan, 1995; Deutsch & Deutsch, 1963; Duncan, 1996; Moray, 1969; Pillsbury, 1908; Reynolds & Heeger, 2009; Treisman, 1964, 1969). You might ask, is it really necessary for the brain to have such a gatekeeper? Can I just let the information from everything that lands on my retina flow into my brain, resulting in recognizing all the objects, surfaces, and details of the scene or movie?

When we look out on the world it feels as though we see everything, but countless experiments have shown that we have a limited capacity to process the representations of objects and surfaces that our visual systems create (Allport, 1989; Amir et al., 2022; Chun et al., 2011; Cowan et al., 2024; Drew & Vogel, 2008; Duncan et al., 2000; Huang et al., 2007; Kahneman et al., 1992; Kanwisher, 1991; Kiyonaga & Egner, 2013; Luck & Vogel, 2013; Martens & Wyble, 2010; Most et al., 2005; Oberauer, 2019; Olivers et al., 2011; Posner, 1980; Pylyshyn & Storm, 1988; Rensink et al., 1997; Robinson et al., 2020; Serences & Yantis, 2006; Simons & Rensink, 2005; Spence et al., 2000; Theeuwes, 2025; Treisman & Gelade, 1980; Wolfe, 2020; Yu et al., 2023). Our intuition that we are simultaneously processing all the objects in our visual field is an illusion. Let us look at some concrete examples.

**Fig. 1:**
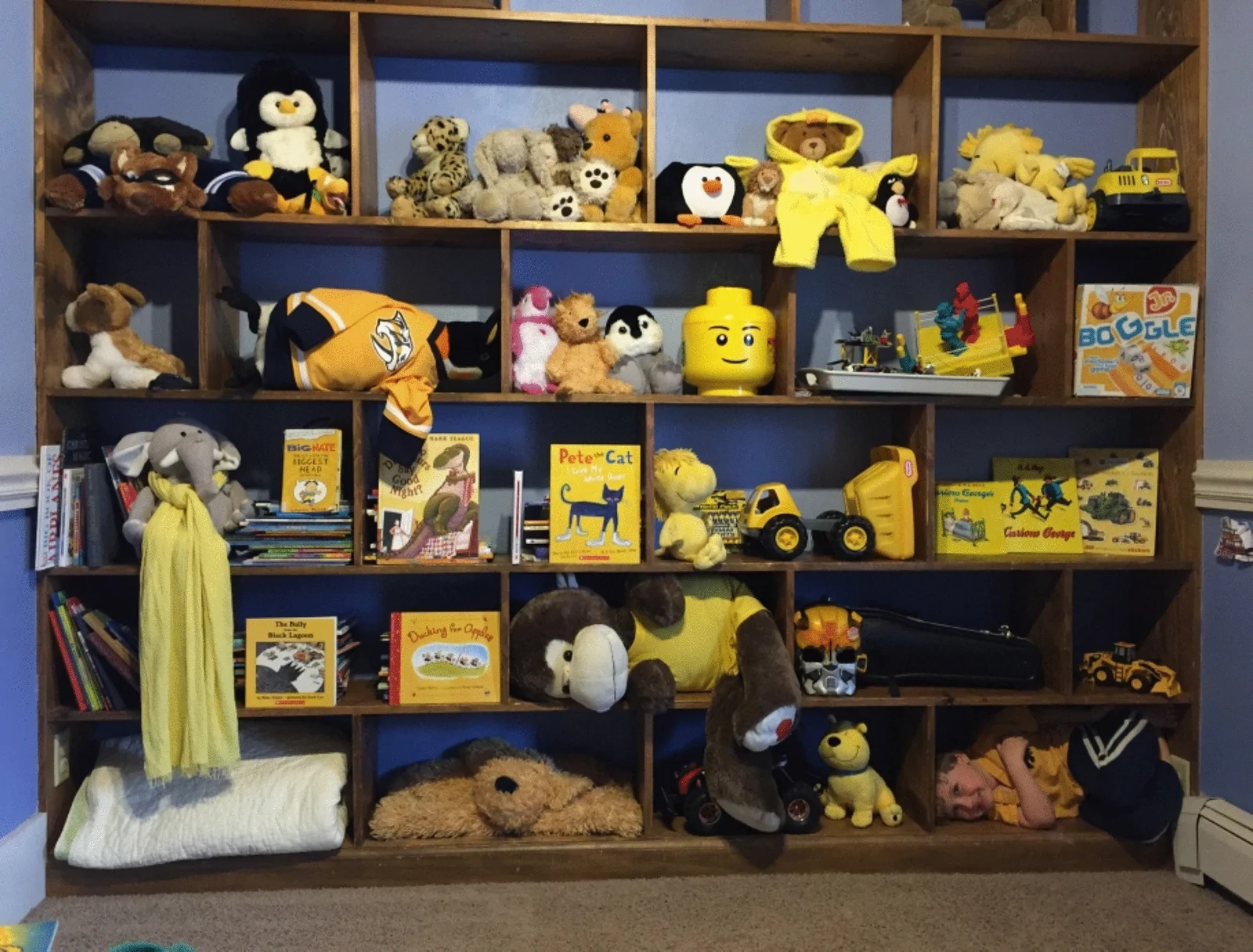
An example of a real-world visual search task; find the child in the yellow shirt playing hide-and-seek

**Fig. 2:**
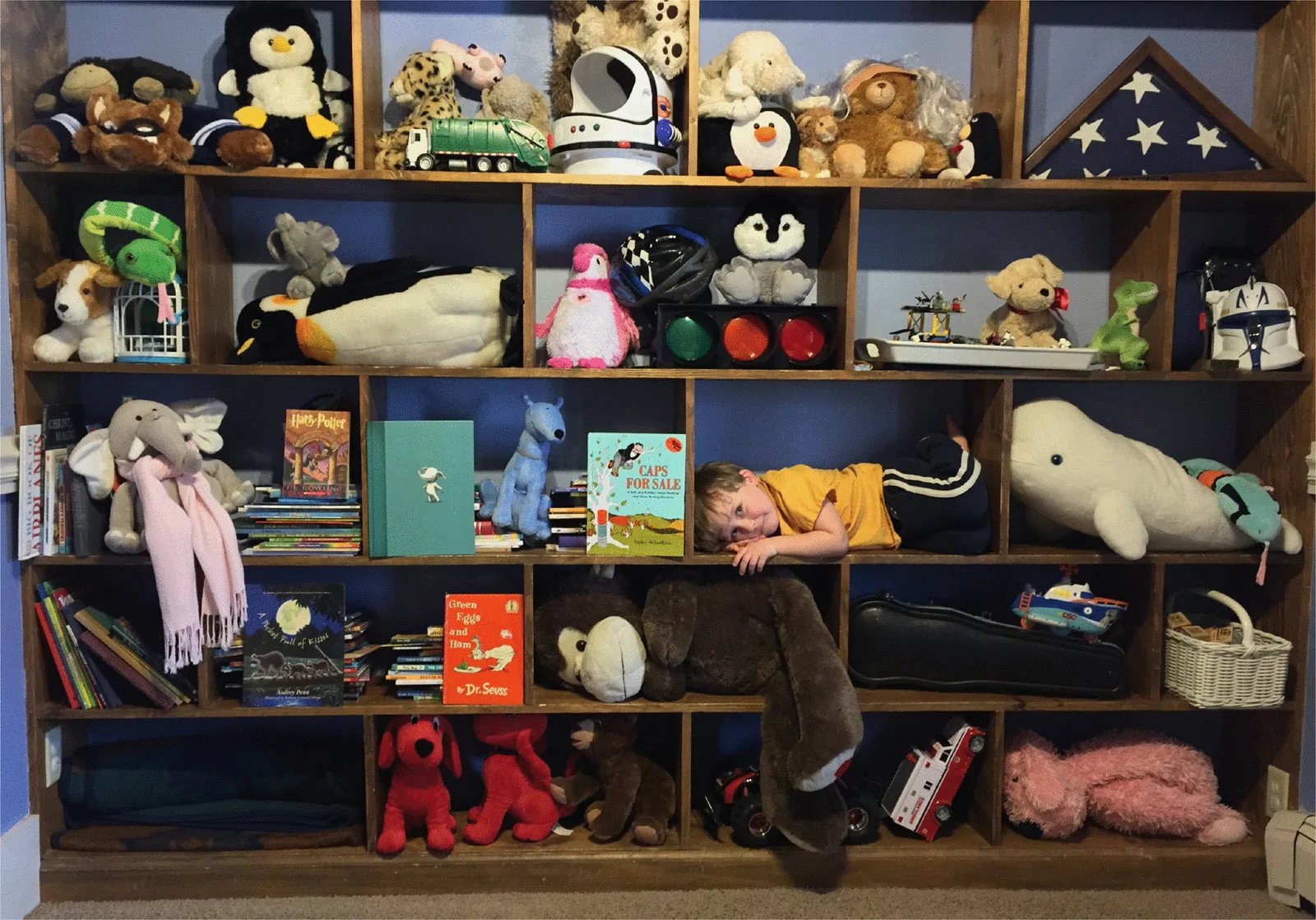
Find the child in the yellow shirt playing hide-and-seek. What makes this hiding place not as good as the scene in Fig. 1? The number of objects that contain the target feature (yellow) is much smaller in this picture than in Fig. 1, so only a few patches of yellow compete for attention in this picture relative to the previous. This is an example of feature-based attentional guidance drawn from the classic literature (Green & Anderson, 1956)

Figure 1 shows a scene from the real world. Find the child in the yellow shirt. Finding your target may have taken you a couple of seconds. This scene has many objects, and many of those objects are yellow. Now, look at the scene in Fig. 2. Finding the target child in the yellow shirt took you less time if you are like typical human participants (Huang et al., 2007; Treisman & Kanwisher, 1998). In the scene shown in Fig. 2, your target did not have to compete for visual analysis with many objects that shared the target-defining feature of yellow (Duncan & Humphreys, 1992). Clearly, we all have a limited capacity to process all of the yellow objects in these scenes. When there are fewer, or even just one yellow object, we can focus visual processing on that object much more quickly. This is the classic finding from the attention literature reported in the first paper using the task called *visual search* that you just performed (Green & Anderson, 1956).

**Fig. 3:**
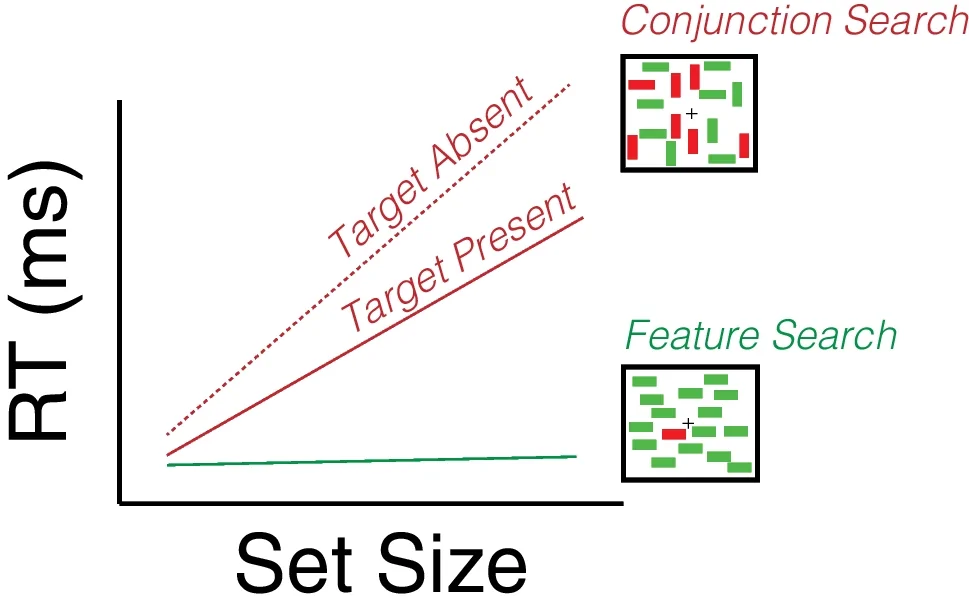
Example of the canonical types of visual search tasks used to study different conditions of search efficiency. Simple feature search tasks show no or minimal effect of the number of distractor objects on reaction time (RT). In contrast, targets defined by a conjunction of features typically evidence slopes of 10–50 ms per object. The target-present arrays are responded to more quickly than target-absent arrays (Treisman, 1988)

Scientists continue to use visual search tasks to study the capacity limitations of visual attention (Wolfe, 2020). These are tasks in which subjects search for a target in an array of objects. The laboratory versions of the tasks are important because they tap into a fundamental cognitive capacity to find targets in our visual field, which is related to our evolutionary fitness (Moon et al., 2022). For example, when we hunt prey, avoid predators, navigate obstacles, search for mates, or watch offspring we are looking for these critical objects and ignoring irrelevant visual information in our environment. Some researchers use real-world images like those in Figs. 1 and 2 (Malcolm & Henderson, 2009; Zsido et al., 2024), but most laboratory experiments use artificial arrays of objects so that the features that define each object can be controlled and manipulated (e.g., see Fig. 3) (Treisman & Gelade, 1980; Treisman & Sato, 1990; Treisman et al., 1977; Wolfe, 1998a, 1998b, 2020). The goal of using artificial stimuli is to isolate the same attentional processes that operate in the visual analysis of the real-world scenes, but while maximizing experimental control over the composition of the stimulus arrays. These artificial stimuli avoid the complications inherent in using real-world scenes where our lifetime of visual knowledge influence their processing (Christie & Klein, 1995; Cohen et al., 2024; Hayes & Henderson, 2022; Intraub, 1997).

The top panel of Fig. 3 shows an example of conjunction search (labeled in red) for a target object composed of just two features, with each of the other objects composed of two features as well. Your task is to find the bar that is red and vertical. Across trials vision scientists vary the number of distractor objects (known as the set size), as indicated with the x-axis label in Fig. 3 (Wolfe, 2020). We can measure how much processing load is exerted by each additional object by measuring your reaction time (RT) to each array. The RT on each trial is the time between when the to-be-reported stimulus is presented and when the participant makes their response (i.e., presses a button, releases a lever, or moves their eye to the target, et cetera). Figure 3 shows how observers’ RTs increase as the set size increases when people search for targets defined by a simple conjunction of two features (Wolfe, 1998a). The slopes of these RT functions allowed researchers to estimate that each additional object requires 10–50 ms of additional processing time (Treisman, 1988; Treisman & Gelade, 1980; Wolfe, 1998b, 2020). This additional processing time with each new object is taken as evidence for a capacity limit in the mind’s ability to process the objects. As the number of objects increases, more time is necessary to process the array. But why does this limit exist?

## Sources of capacity limits: Neurons, receptive fields, and feature selectivity

What are the sources of our capacity limits? Our visual system is faced with unique computational challenges when presented with arrays of objects (Marr, 1982; Moran & Desimone, 1985; Mozer, 1991). The neurons in our visual systems respond to stimuli within a certain region of space. This is known as the neuron’s receptive field based on the idea that it receives inputs from that subfield of space (Huble & Wiesel, 1968). Neurons in the visual system typically have what is known as selectivity, in that they become tuned to certain features or combinations of visual features and fire maximal bursts of action potentials when those features are present in the receptive field (e.g., yellow, a vertical bar, a yellow vertical bar, a 45° right-titled 4-cycle per degree Gabor, etc.) (Palmer, 1999). These neurons can clearly signal what is present in its receptive field when an illuminated object appears against a black background, as it does in vision science experiments of object perception or memory in the laboratory (Cao et al., 2025; DiCarlo et al., 2012; Logothetis & Sheinberg, 1996; Rainer et al., 1998). However, when we present an object embedded in an array of objects, as they typically exist in our ambient environment, the responses of the neurons to the stimuli in its receptive field can become ambiguous (Luck et al., 1997b). Specifically, the responses of individual neurons become ambiguous when more than one object appears in the receptive field of a neuron, such that downstream neurons could not use the neuron’s responses to decode what the upstream neuron was seeing (Moran & Desimone, 1985; Mozer, 1991; Reynolds & Desimone, 2003). It may be exactly this form of neuronal interference that attention is seeking to mitigate when faced with the task of accurately processing the information from cluttered visual arrays (e.g., Luck et al., 1997b; Reynolds & Chelazzi, 2004).

**Fig. 4:**
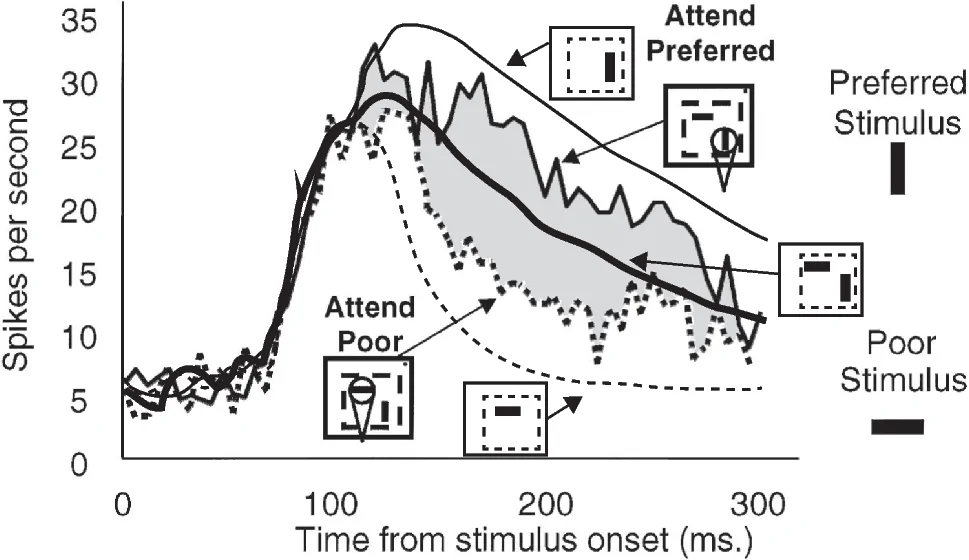
Summary of attention effects from recordings of neurons in V4. The circle signifies the attended stimulus in the cell’s receptive field (marked with the dashed box). The figure shows that when attention is focused on a stimulus, whether it is the preferred stimulus that elicits higher firing rates or the poor stimulus that elicits lower firing rates in this neuron, the response of the neuron comes to be dominated by the attended stimulus. For example, note how the neurons most vigorous response is when just the preferred stimulus is in the receptive field (shown with the thin solid line) and the weakest response is when just the poor stimulus is presented (shown with the thin dashed line). The thick solid line in the middle of the functions is the neuron’s response when both the preferred and the poor stimulus are simultaneously presented in the visual field, without attention being deployed to one of the stimuli. The key effects of attention are shown in the medium weight lines with the solid line showing the mean firing rate at each moment when the preferred stimulus was attended. The medium weight dashed line shows this same neuron when the poor stimulus was attended. In both of the attention conditions the effect of focusing attention is to make the neuron signal the features of the attended stimulus in its receptive field, filtering out the effect of the distractor (e.g., through active suppression or input gating). Reprinted with permission from Reynolds and Desimone (1999)

Imagine a neuron in area V4 of the visual system of a primate brain. It is wired to lower-level neurons in V1 and V2 such that it responds vigorously to a red vertical bar (i.e., with a high firing rate), but respond weakly to a green horizontal bar (i.e., with a low firing rate). However, its receptive field is about 5° of visual angle wide. This is wide enough that when an array of objects is presented, multiple objects fall in its receptive field, as illustrated in Fig. 4. How vigorously will it respond to the objects in its receptive field?

Experiments with macaque monkeys have shown that when the two objects shown in Fig. 4 appear in the neuron’s receptive field, it responds with a firing rate that approximates the average of its responses when each object appears in its receptive field in isolation (Reynolds & Chelazzi, 2004; Reynolds & Desimone, 2003; Reynolds & Heeger, 2009; Reynolds et al., 1999). If a downstream neuron was listening to our neuron’s firing rate to determine which visual features were present in its receptive field (red vs. green, and horizontal vs. vertical), it could not decode which features were present. The stimulus in the world could be green and vertical, or red and horizontal, or all of these.

This is an example of the classic *binding problem* (Wolfe & Cave, 1999). As we know from basic vision science (Grill-Spector & Malach, 2004), different visual features such as color, motion, or shape, appear to be processed in different visual areas of the brain (Van Essen & Maunsell, 1983; Zeki, 1978; Zeki et al., 1991). At the level of the individual neuron, the problem is that neurons are tuned to specific features or combinations of features (e.g., red and vertical), with different neurons processing different features (i.e., red vs. green, or color vs. motion). It is as if we have all these independent little machines that build different pieces of a product in our brains. The question posed in the statement of the binding problem is the following.

How do we put the feature pieces back together to form the objects that we perceive? Alternatively, if we have all these different areas and neurons processing different features, then how do we correctly bind features when multiple objects are presented in our field of view? One of the key roles of attention in the visual system appears to be to bind together features of an attended object (Treisman & Schmidt, 1982), allowing neurons that code for its features to unambiguously signal the presence of the critical object to the rest of the brain (Bernstein & Robertson, 1998; Buffalo et al., 2010; Kastner et al., 2001; Mitchell et al., 2009).

Returning to our example V4 neuron, we can make one of the two objects in its receptive field attended by having that particular object be the target of your brain’s search task. We can do this by having the monkey receive a drop of juice for releasing a lever to report that the target is present in the array, or by having the monkey make an eye movement to the target in the array (Chelazzi et al., 1998, 2001; Reynolds & Chelazzi, 2004; Reynolds & Heeger, 2009). Now, the neuron responds very differently from the case in which both objects are present in its receptive field, but unattended (i.e., Fig. 4, thick middle trace). If the monkey is searching for the red vertical bar, then the neuron responds with a high firing rate, as if that attended object is the only object that appears in the receptive field. If the green horizontal bar is the target of the monkey’s search, then the neuron responds with a low firing rate, as if that attended object is now the only object in the receptive field. That is, when attending to an object in the visual field, attention appears to filter out the ignored distractor stimuli (Moran & Desimone, 1985). Findings like these support the hypothesis that one of the primary roles of attention in the visual system is to solve the binding problem by selecting individual objects so that the neural responses in the visual system unambiguously signal the features of that attended object (Treisman, 1999).

In recent decades researchers have proposed a competing hypothesis that brain oscillations solve the binding problem by linking neurons with a temporal code (Lisman, 2005; Lisman & Idiart, 1995), thus binding together arbitrary pieces of information (Singer & Gray, 1995; Steinmetz et al., 2000). This is an attractive hypothesis that provides an explanations for what seem to be the inherent oscillatory dynamics of electrical brain activity (Adrian, 1944; Adrian & Matthews, 1934; Nunez & Srinivasan, 2006). Although, how such a signal could be used or read out from the brain without being connected to all of the possible neurons is not clear (Shadlen & Movshon, 1999).

## Shifting attention

**Fig. 5:**
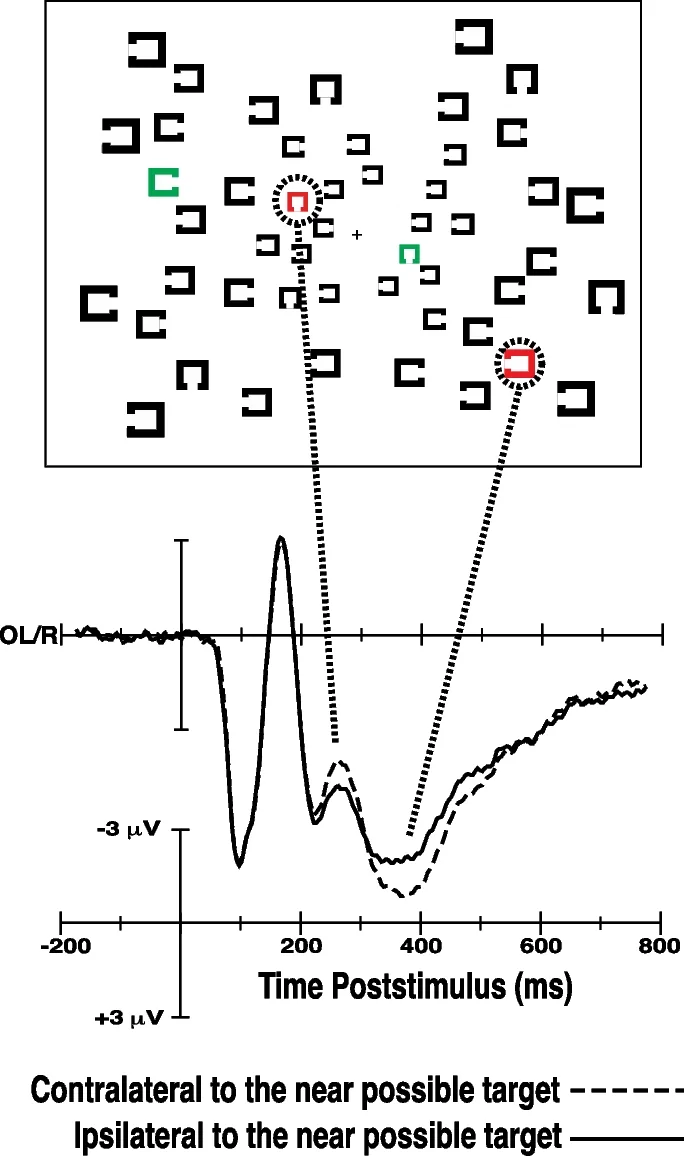
The stimuli and recordings of event-related potentials (ERPs) showing attention bounce between possible targets during visual search. Reprinted from Woodman and Luck (2003a, b) with permission from APA and myself

Theories that account for how features are bound together typically propose that attention focuses on one object at a time when we view the scenes in Figs. 1, 2, 3 (Treisman, 1996; Treisman & Gelade, 1980; Wolfe et al., 1989; Woodman et al., 2022; Zhang et al., 2019). This is proposed for several reasons. First, proposing that attention focuses on individual objects and shifts between them can explain the increasing RTs as set size increases (Wolfe, 1998a). If we assume that attention shifts between individual objects and each shift takes some time (e.g., 50 ms), then when there are more objects, more shifts are required, and the RT should increase (e.g., by 50 ms with each object). Second, electrophysiological studies of both humans and macaque monkeys show that we can measure attention as it shifts between possible target objects in visual search arrays (see Fig. 5) (Bichot et al., 2005; Bisley & Goldberg, 2010; Motter & Belky, 1998; Woodman & Luck, 1999, 2003b). Third, shifting between objects one at a time allows for unambiguous responses of neurons in high-level areas of the visual system where receptive fields are large and can include an entire visual hemifield (e.g., the left side of the visual field) (Bichot et al., 2001; Bisley & Goldberg, 2003; Felleman & Van Essen, 1991).

**Fig. 6:**
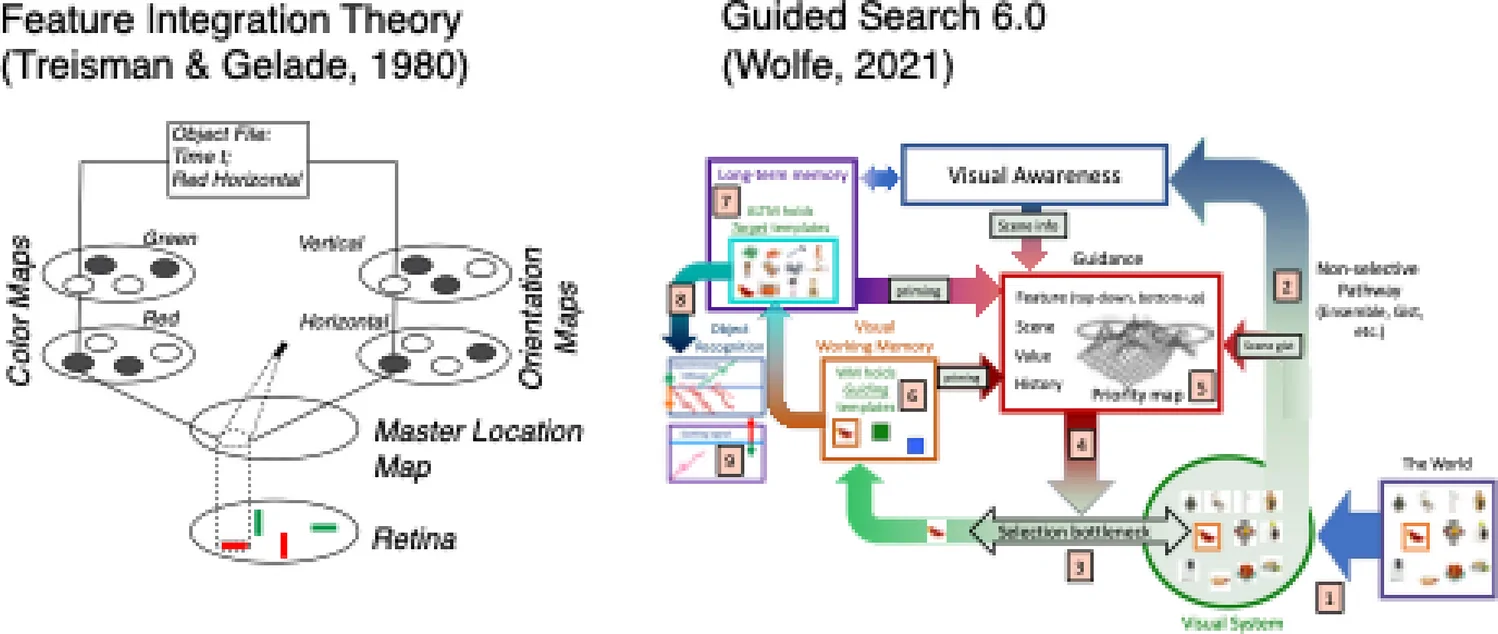
Feature integration Theory from 1980 is shown on the left and Guided Search from 2021 is shown on the right. The models are related in that they both propose pre-attentive feature processing and serial processing due to shifts of attention. However, the elaborations necessary to account for visual search performance are evident in the additional complexity of the more modern model

Figure 6 shows an illustration of two models of what your brain did when performing the visual search tasks in Figs. 1 and 2. The model on the left is the early Feature Integration Theory of Treisman and colleagues (Treisman, 1988; Treisman & Gelade, 1980; Treisman & Sato, 1990). The more complex model on the right, the Guided Search 6.0 model of Wolfe (2021), represents the state-of-the-art offspring of the ideas of Feature Integration Theory, but with the complexity needed to account for the diversity of findings from the now large and growing visual search literature (Anderson et al., 2021; Carlisle & Woodman, in press; Theeuwes, 2025; Wolfe, 2012; Wolfe., 2020; Wolfe et al., 2016). The additional complexity of the newer model demonstrates how our understanding of the sophistication of the mechanisms has improved, with the current model describing roles played by awareness, object recognition, working memory, long-term memory, and cognitive control mechanisms of the brain to account for how our brain processes visual search arrays.

After the activity from the transduced input leaves the retina, it is split into processing streams that already specialize in the processing of different types of visual information (Palmer, 1999). As that information is propagated through the visual system, it is passed through two different neuroanatomical processing streams that begin in the retina in the form of different sized retinal ganglion cells (Goodale & Milner, 1992; Milner & Goodale, 1993; Mishkin et al., 1983). One is a fast dorsal stream which specializes in the processing of spatial and motion information, and the second a slower ventral stream which processes object features such as color and shape (Ungerleider & Mishkin, 1982). This results in different visual features being processed by different neural circuits in the visual system. However, this information needs to be reintegrated to form the unified visual percepts of objects and surfaces that we experience when we look out on the world (Beck et al., 2001; Hommel & Colzato, 2004; Rao et al., 1997; Roelfsema et al., 1997). As discussed next, the control of attention involves combining these multiple types of information in high-level areas of visual cortex.

## Controlling attention

I have just discussed the idea that when we look out on the world, we shift attention to certain inputs to our visual system. What determines which inputs are selected by attention?

Researchers agree that at least two types of factors determine whether an object will be selected: bottom-up factors and top-down factors (Anderson et al., 2021; Awh et al., 2012; Buschman & Miller, 2007; Ekstrom et al., 2008; Kinchla & Wolfe, 1979b; Theeuwes, 2025; Vecera & Farah, 1997; Wolfe, 2021). Bottom-up factors are the basic features of the object that make it stand out from its surrounding context (this is also often known as *salience*) (Donk & Theeuwes, 2003). For example, a yellow object in a sea of blue (as in Fig. 2) creates a local heterogeneity in the visual field that appears to summon attention to itself, as I describe next.

Bottom-up factors refer to the fact that the information the visual system needs to determine that an item is special (that is, the yellow item in a sea of blue) is extracted in the lowest levels (i.e., bottom) of the visual system (such as area V1 of the visual system) (Felleman & Van Essen, 1991). Indeed, some visual attention researchers describe this as a *pop-out search array*, consistent with subjects’ experience that items seem to “pop-out” from the array (Nakayama & Joseph, 1998; Nothdruft, 1991). In these situations, the target object is so different from the objects and surfaces around it that we appear to automatically shift attention to these objects to process them, as inferred using subjects’ RTs to such search targets (Jonides, 1981; Theeuwes, 2025).

Bottom-up factors can make subjects’ behavioral responses to these pop-out targets fast and independent of the number of objects that appear in the same array or scene (i.e., independent of the set size of the search array) (Jonides & Yantis, 1988; Yantis & Jonides, 1990). Moreover, if bottom-up factors draw attention to an object that is not the task-relevant object in the search array, then subjects’ RTs are slower compared to when they search for a target in the absence of this pop-out item (Remington et al., 1992; Theeuwes, 1994; Yantis, 1993).

## Attentional capture

**Fig. 7:**
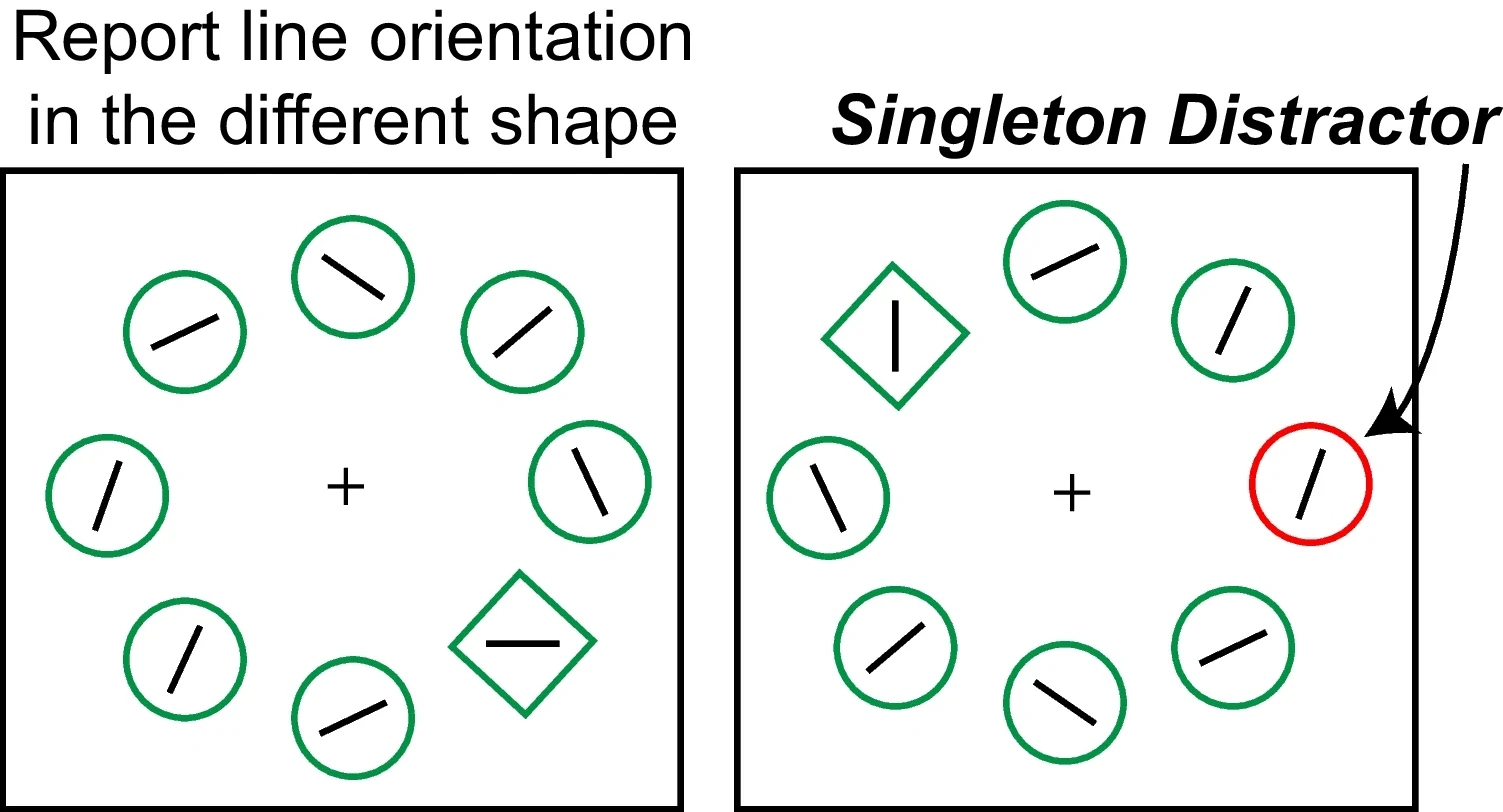
In this visual search task, the observer is supposed to find the different shape (a diamond in this case) and report the line orientation (i.e., horizontal or vertical). The color singleton is irrelevant for this task. But when it is present, it slows observers’ responses to the task-relevant information in the array (i.e., the shape singleton which defines the target). This is the phenomenon referred to as attentional capture (Anderson et al., 2021; Theeuwes, 2025)

It appears that some events attract attention to themselves, even when we want to ignore such things. This phenomenon is known as *attentional capture* and was first discussed as a unique result of the sudden onset of an object appearing in the visual field (Jonides & Yantis, 1988; Remington et al., 1992; Yantis & Jonides, 1984). However, studies showed that unique elements in otherwise homogenous arrays (i.e., a red object in a field of green objects, see Fig. 7) could also appear to involuntarily capture attention. Similar effects have been found with motion, orientation, and other visual features demonstrating that it is unique items in our visual field that have strong advantages when competing for attention, not just objects that suddenly onset (Folk & Annett, 1994; Mounts, 2000).

A significant shift in the study of attentional capture occurred after researchers suggested that the strongest attentional capture comes from objects that match the features of the target you are looking for (Folk et al., 1992). Subjects can efficiently search for a green target, even when task-irrelevant white dots appear in the periphery. However, on some trials, some of the dots in the periphery were the target color (i.e., green), and on these trials people were slower to find the target. The idea is that the target matching input caused a shift of attention to its location, slowing attention to shift away to the actual target location. This type of attentional capture also appears to occur when participants view streams of stimuli and target-colored items appear in the stream (Folk et al., 2002).

Making a further case for the importance of top-down control regarding whether our attention is captured, other researchers have proposed that the strategy that participants use to perform visual search changes whether attentional capture is observed. Specifically, Bacon and Egeth (1994) proposed that participants engage in one of two different modes of controlling attention during the capture tasks shown in Fig. 7. They proposed that participants either engaged in feature-search mode or singleton-detection mode. During feature-search mode subjects search for the specific feature that defines the target, instead of any unique (or singleton) object because on some trials the target is not the most salient item. This mode of processing prevents attentional capture by the irrelevant but highly salient singletons. In contrast, during singleton-selection mode, subjects search for any salient or unique item because the target is always salient. This allows attention to be captured by any unique object in the visual field because it matches the thing the participants are looking for (Drennan & Gaspelin, 2026; Lamy et al., 2006; Leber & Egeth, 2006).

As I will return to later, current debates continue to contrast the effects of goals with the competing perspective that attentional capture is entirely driving by low-level visual salience (Anderson et al., 2021; Awh et al., 2012; Donk & Theeuwes, 2003; Theeuwes, 1995, 2025; Wang & Woodman, 2024). The general idea that bottom-up factors can control attention is contrasted with top-down factors that make the representations of certain objects the processing goal of our visual system.

In contrast to bottom-up factors, top-down factors that influence attention are those that designate an item as being relevant for the task we are performing, regardless of its bottom-up salience (Baluch & Itti, 2011; Cavanagh, 1991; Hopfinger et al., 2000; Kinchla & Wolfe, 1979a; Westerberg et al., 2023). We just heard about how top-down factors can result in items capturing attention (Folk et al., 1992), although we believe the use of top-down control of attention is common in the real world. For example, a basketball player who is trying to shoot a basket must focus attention on the small stationary rim of the basket as the opposing fans jump, scream, and wave their hands and signs. This type of attentional control was critical when our ancestors spent their days hunting animals that were well concealed in their environment.

The top-down control of attention is believed to be dependent on our memory representations (Gunseli et al., 2014; Pillsbury, 1908; Woodman & Chun, 2006; Woodman et al., 2013). Computational models of attention focus on the role of visual working memory representations in controlling attention (Bundesen & Habekost, 2008; Bundesen et al., 2005; Hamker, 2004). This is because of the seminal work we discuss next in which recording neuronal activity in monkeys showed that neurons in the temporal lobe maintain high levels of activity following the presentation of a target cue while the screen is blank and the monkey is waiting to search for that target in an array of objects (Duncan, 1996, 1999). This elevated firing rate is taken as evidence that the features represented by the neuron are being held in an active state in working memory. Consistent with this idea, research suggests that humans also hold representations of targets in visual working memory when learning a task or when targets frequently change (Carlisle et al., 2011; Schneider & Shiffrin, 1977; Shiffrin & Schneider, 1977; Soto et al., 2005; Woodman et al., 2007). Sometimes we maintain representations that help our visual systems discriminate between targets and distractors, even if it means maintaining a target representation of a color or feature that is not exactly the target, but instead helps separate distractors from the target (Scolari et al., 2012; Yu et al., 2022, 2023).

Measuring neural activity as primates perform visual search tasks has been critical in revealing how the brain provides top-down attentional control and guides attention to the targets that we seek (Buschman & Miller, 2007, 2009; Chelazzi et al., 1998, 2001). Psychologists from the beginning of the last century proposed that memory representations determined what we focused attention on (Pillsbury, 1908). But how can we observe those memory representations controlling the visual system?

## Attentional control using memory

**Fig. 8:**
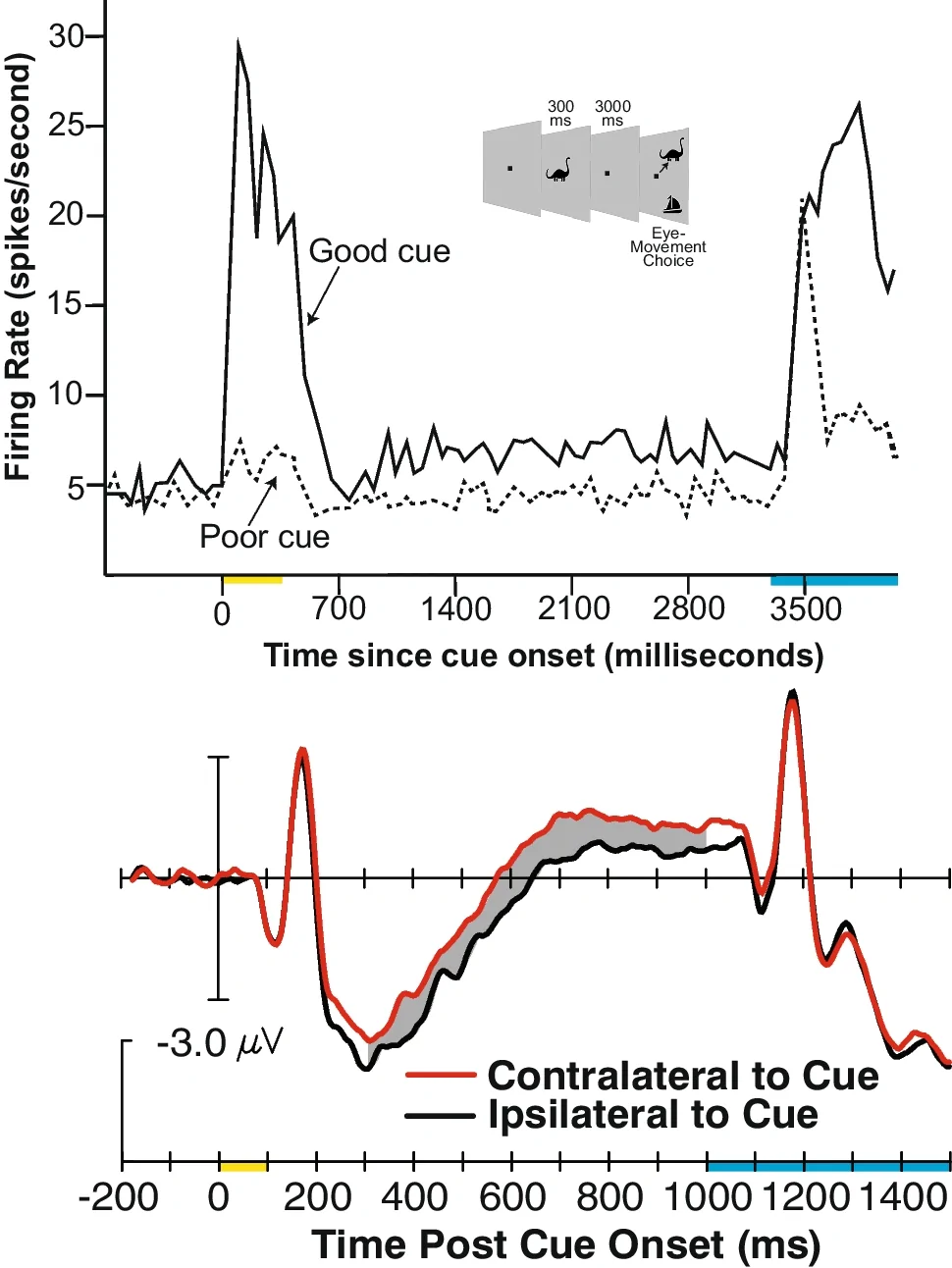
Findings from cued visual search experiments with monkeys (top panel, recording from Chelazzi et al., 1993) and humans (bottom panel, grand average from Carlisle et al., 2011). In both plots 0 ms marks when the cue appeared showing the primate what to look for and the shading on the time axis shows when the cue (yellow) and search array (blue) were presented

Chelazzi and colleagues (1993) developed an elegant method for studying the control of attention by memories. They cued macaque monkeys to search for a particular object, then the search array was presented about 3 s later (see Fig. 8, top panel). The monkeys had to signal whether the target was present to receive a juice reward. They recorded from neurons in the temporal lobe that would respond most vigorously to certain objects in the set of possible targets. What they observed was simple but had important implications.

The top panel of Fig. 8 shows that they found that neurons selective for a target objects would exhibit an elevated firing rate between the presentation of the cue and the onset of the search array (Chelazzi et al., 1998). This elevated activity is consistent with the brain working to maintain a working memory representation of the target it was about to search for (Desimone, 1996). That is, the neuron is driven by the presentation of the good stimulus as a cue. This *good cue* spike density function shows a large sensory response followed by a sustained elevated spike rate effect which is the difference between the solid and dashed lines from ~ 700 ms postcue onset to the onset of the search array at ~ 3,000 ms postcue onset. This higher firing rate is believed to be due to the cue being held in working memory during the interval prior to search. Thus, when we want to find our car in parking lot, we simply maintain a representation of it in working memory. We could also imagine how holding a representation of red in working memory allows us to implement feature-based attention, attending to all red items (Hollingworth & Bahle, 2020; Liu, 2019; Olivers et al., 2006; Zhang & Luck, 2008).

Several decades later, Carlisle and colleagues performed fundamentally the same type of experiment with human subjects (Carlisle et al., 2011). They cued subjects to look for an object in a search array. For their human subjects they used similar square stimuli instead of the complex pictures used to drive temporal lobe neurons in the studies of Chelazzi and colleagues. Carlisle and colleagues measured a component of the subjects’ event-related potential (ERP) waveforms known to index the maintenance of information in visual working memory (Luria & Vogel, 2011; Luria et al., 2016; Vogel & Machizawa, 2004; Vogel et al., 2005a). This signal is known as the contralateral delay activity, because just as the visual system has a contralateral organization. That is, inputs from the left side of space are initially routed to the right hemisphere, and inputs form the right side of space are routed to the left hemisphere. It appears that the working memory activity maintains this lateralized organization. As shown in Fig. 8, they observed that this ERP waveform was clearly present in the interval between the target cue and the search array, just like what was found in macaque monkeys performing visual search (compare the activity while the screen is blank in the bottom panel of Fig. 8 to the top panel).

In the experiments that I just discussed, the target changed from trial-to-trial, but what happens when we look for the same object for minutes or hours continuously? For example, if you are a baggage screener working at an airport, do you really hold representations of the weapons you seek actively in working memory hour-after-hour, performing the same task across days and months?

When people look for the same thing for many minutes it appears that the brain may be able to utilize long-term memories to control attention. Carlisle and colleagues found that when the same target was cued across multiple trials in a row, they could watch the visual working memory activity disappear within about five trials of searching for the same object (Carlisle et al., 2011; Reinhart et al., 2014). This was observed as subjects were becoming faster at searching for that target repeatedly (Reinhart et al., 2014). They inferred that this was evidence that long-term memory representations were taking over the top-down control of attention from visual working memory. Subsequent analyses and experiments revealed ERP effects related to long-term memory retrieval that supported this theoretical proposal that long-term memory representations take over attentional control from working memory representations (Logan, 2002; Pillsbury, 1908; Shiffrin & Schneider, 1977).

**Fig. 9:**
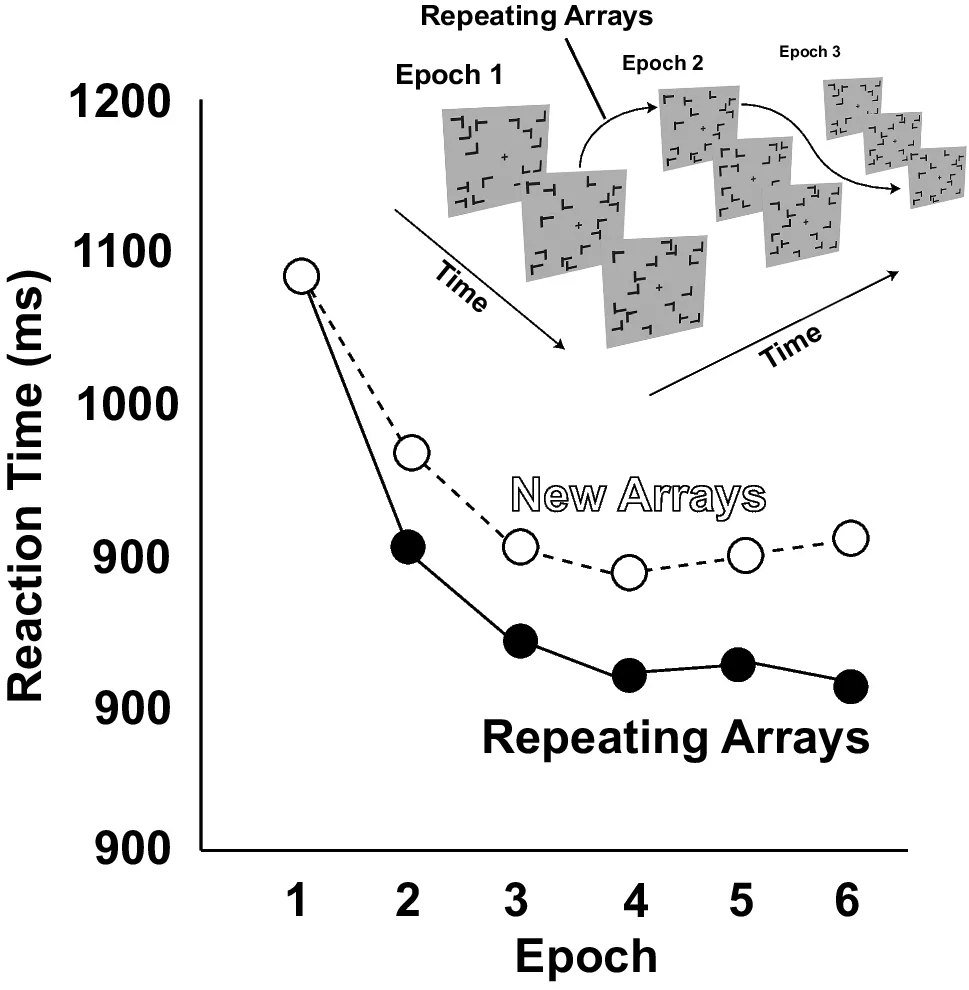
Illustration of the canonical spatial contextual cuing phenomenon. In Chun and Jiang (1998), observers were asked to press buttons indicting whether the T in the array was pointed to the left or the right. Unbeknownst to the subjects, some of the arrays of distractors repeated each epoch (i.e., block of trials) as shown with the inset diagram. Even though subjects were not aware of these repetitions, and could not recognize the repeated arrays, they were faster at finding the Ts in these arrays, as shown in the unfilled data points in the graph. The filled circles show search speed in randomly generated new search arrays. Although subjects show a general speeding of responses as they practice this task, the repeated arrays provide a context that cues attention to the target locations as these contexts are learned across the experiment

Figure 9 shows an example of findings from a *contextual cueing* experiment (Chun, 2000; Chun & Jiang, 1998, 1999). In these tasks subjects perform visual search tasks, similar to those we have been discussing. However, interleaved with the randomly generated novel arrays of distractors are repeating arrays of distractors. Even after a single presentation of one of these repeated arrays, subjects can find targets more quickly than in novel arrays of distractors (Chun & Jiang, 1998). These effects appear to last for weeks, because if you have subjects view these repeated arrays, they are still faster when looking for targets again even up to a month later (Chun & Jiang, 2003). The duration of this effect allows us to conclude that this information about these repeated arrays must have been stored in long-term memory. This effect is diminished in patients with amnesia, providing other evidence of long-term memory storage of this contextual information (Chun & Phelps, 1999). This long-term memory effect is particularly striking because observers cannot report having seen the repeated arrays before, demonstrating that even implicit long-term memory representations can provide top-down control of visual attention.

The idea that attention is guided to objects that match the representations we have in memory is powerful in explaining phenomena in the attention literature. Many studies have examined *feature-based attention* (Maunsell & Treue, 2006; Schoenfeld et al., 2007; Shih & Sperling, 1996; Treue & Martinez Trujillo, 1999; Zhang & Luck, 2008). Feature-based attention refers to the idea that the visual system can be biased to select objects or surfaces having a certain feature (e.g., yellow, as in our examples from Figs. 1 and 2), regardless of where it appears in space.

Zhang and Luck (2008) showed an example of how the human brain exhibits attention effects to peripheral red stimuli when looking for a red target, even when those red stimuli appear at locations that are distant from the expected target location (i.e., in the other visual hemifield). Research suggests that we can control which features will capture attention by having an *attentional set* that consists of the task-relevant features stored in memory (Most et al., 2005; Treisman, 1969; Wang & Woodman, 2024). Objects that do not match the attentional set are often missed when participants are later asked if they saw them, resulting in an effect known as inattentional blindness (Most et al., 2005).

The idea is that we set our visual systems to be particularly sensitive to the features of the targets that we need to process for the task at hand. As a result, bottom-up salience alone does not appear to determine which inputs attention becomes focused on (Atchley et al., 2000; Folk et al., 1992, 2002; Gibson & Kelsey, 1998). Instead, the critical component is whether a feature like color, shape, or sudden onset is stored in memory as the target feature we are looking for in our visual field (Folk et al., 1994). Thus, it seems that whether an input to the visual system is selected by attention is a function of both its bottom-up features and the memory representations that determines the top-down attentional settings required for the task that the subject is performing. Later I return to this issue and discuss the brain areas that appear to integrate this bottom-up and top-down information.

The evidence I just discussed demonstrating that attention can be biased to objects with certain features (i.e., feature-based attention) will be contrasted with visual-spatial attention in the next section. However, it is useful to return to the real-world examples that we began with. Frequently, we use our visual systems in our daily life to find certain objects, such as a contact lens on a floor or a child on a busy playground, thus, our ability to bias our visual systems to preferentially processes these targets is a critical ability that we use so frequently that we often take it for granted.

## Spatial cueing of attention

If we can bias attention to select objects with specific features, then it seems natural that we should be able to directly guide attention to certain locations or regions of space. After all, spatial location is a feature of the objects we see when we look out on the world.

**Fig. 10:**
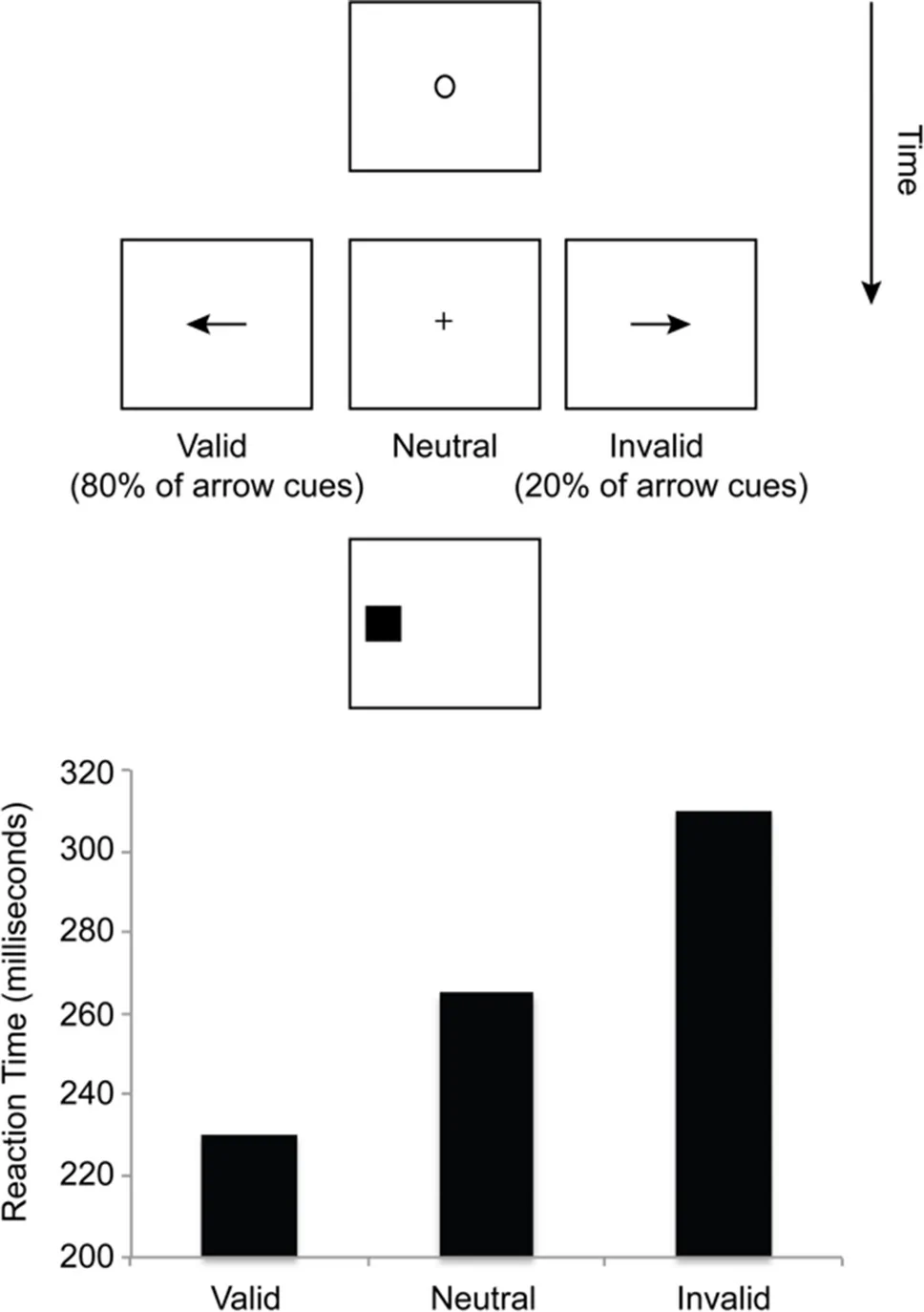
A paradigm used to cue attention to spatial locations. The top panels show how the task unfolds. Half of the trials are neutral in which no spatial cue is presented. Of the other half of trials, 80% have arrows pointing to where the target will appear a second later. Cues to the left and right were equally probable, with most of them being valid (80%). Adapted from Posner, Nissen, and Ogden (1978) and Posner et al. (1980)

Try it. Look to your left. This is an example of using a *spatial cue* to signal where a target might appear (Posner, 1980). A spatial cue is any signal that directs attention to a spatial location in advance of a task-relevant stimulus. The spatial cue could appear at the location of the upcoming stimulus as a box appearing around the possible target location or the cue could be symbolic, like the arrows shown in Fig. 10. As you might imagine, when we present a spatial cue indicating the target location, subjects can respond to the targets that appear at that location more quickly than when the same target appears at a different location.

When I told you to look left, you probably moved your eyes and maybe even turned your head to the left. These gross muscle movements are known as *overt attentional orienting* (Posner & Cohen, 1984). That is, you made overtly observable changes using your muscles so that the high-resolution fovea would be pointed toward the left. Studying overt attentional selection in the laboratory is straight forward. You measure where people direct their gaze using a camera pointed at the eyes, with most modern eye trackers using infrared light in these cameras (Fischer & Breitmeyer, 1987; Henderson, 1993; Hoffman & Subramaniam, 1995; Liversedge & Findlay, 2000; Trentin et al., 2023; van Ede et al., 2019). But eye tracking is not the only way to study attention. Indeed, it appears that attention works to select objects even when the eyes are not moving.

If we tell you to be prepared for a target on the left without moving your eyes or your head, your responses to it will be faster than if we do not tell you where the object will appear. This is due to *covert attentional orienting*, where you do not move your eyes or head, but show evidence that you have shifted attention to a location covertly (Carrasco & McElree, 2001; Eriksen & Eriksen, 1974; Posner et al., 1980, 1987). Let’s see how this covert attentional selection can be measured.

Hermann von Helmholtz was an early pioneer in cognitive science, particularly vision science, as he was the originator of the tri-chromatic theory of color vision (Helmholtz 1866). Many also consider him to be the first vision scientists to study covert attention (Helmholtz 1896). Unlike current scientists who enjoy the luxuries of computer technology, he built his own experimental apparatus to run the experiments to convince himself that covert attention (i.e., keeping the eyes fixed at one location, while trying to process stimuli at a different location) was an ability that he possessed (Helmholtz 1896).

He built a wooden box with two pinholes so that each eye could see into the box. Objects were then placed in this box that was completely dark at the beginning of the experiment. While keeping his gaze fixed, Helmholtz then directed his attention to a particular region of his visual field. A spark was then used to briefly illuminate the box for an instant. He reported that he could make out the objects in the region he had been attending to but was unable to recognize the objects elsewhere. As far as we know, this was the first reported demonstration that attention could be deployed independently of eye position and accommodation.

The laboratories of Charles Eriksen and then Micheal Posner pioneered the use of cueing paradigms to study covert attention (Eriksen & Collins, 1969; Posner, 1980). In the initial experiments with spatial cueing in the modern era, researchers contrasted the speed with which subjects could respond to targets at a cued location (known as a valid cueing trial) with responses when the cue and the target appeared at different locations (known as an invalid cueing trial) (Eriksen & Collins, 1969). The idea is beautifully simple. If we draw your attention to a location with a cue, then attention is already there when the target appears moments later. This results in faster responses to that target. However, if the target appears at a different location than marked by the cue, attention needs to be shifted through space to the target, slowing its processing and the ultimate behavioral response. From results like these, we do not know whether we are measuring the benefit of having attention at the target location, or the cost of shifting attention to the target from the invalidly cued location. To determine whether we were observing costs or benefits of attention, a better baseline was required.

The reason that the spatial cueing paradigm has become known as the *Posner cueing paradigm* is that Michael Posner and his colleagues had the insight that we could separately measure costs and benefits associated with attentional selection if we had a neutral baseline with which to compare our findings from valid and invalid cueing trials (Posner, 1980; Posner et al., 1980). Figure 10 shows the stimulus conditions used to implement spatially valid, invalid, and neutral cueing trials, along with the classic behavioral findings used to separately measure the costs and benefits of focusing attention on locations. The neutral trials essentially provide no information about whether the target will appear at the left or the right. As a result, we can see that invalid cues hurt the performance of the subjects in terms of speed (i.e., slower responses to the targets), relative to getting no information on neutral cue trials. This provides a measure of the cost of having attention misdirected. On the other hand, we see that valid cues improve the speed (i.e., faster responses to the targets) of subjects relative to getting no information about the target location on neutral cue trials (Posner, 1980).

## Dynamics of visual-spatial attention and inhibition of return

It was proposed that the patterns of behavioral performance shown in Fig. 10 allow us to infer the movement of a spatial spotlight of attention (Posner & Cohen, 1984; Posner et al., 1980). This metaphor continues to provide attention researchers with an intuitive way of thinking about what attention might be doing in the visual system and is consistent with the initial ideas of Helmholtz regarding attention increasing the visibility of a stimulus. Providing a cue at a location allows the spotlight to become focused there, illuminating the stimuli so that they appear higher in contrast (or brighter), and can be processed more efficiently by the visual system (Carrasco et al., 2004). By efficient processing, researchers mean that visual inputs selected by attention can be more quickly or accurately processed relative to items not within the spotlight of attention. Because of the elegance of this metaphor, logic, and method, the Posner cueing paradigm has been a dominant method used to study *visual-spatial attention* for nearly 40 years (Lu & Dosher, 2023; Trost & Gibson, 2024).

**Fig. 11:**
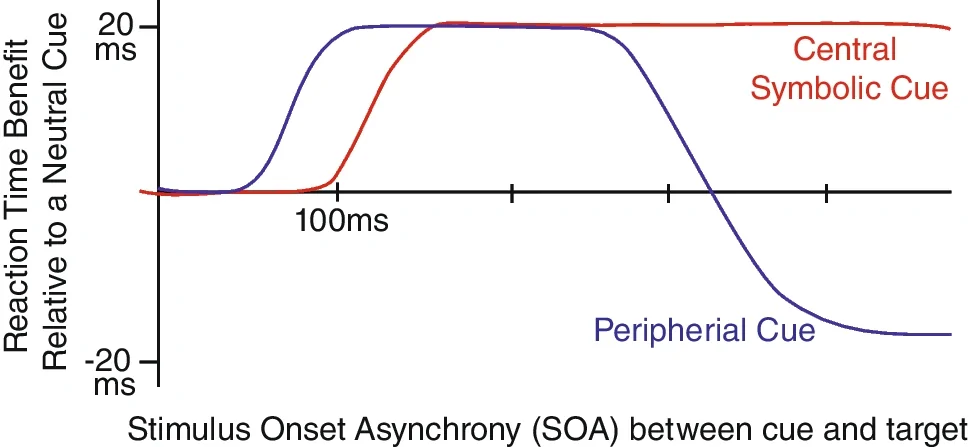
Illustration of how spatial cuing effects develop across time. Peripheral cues have an effect earlier, but then this effect reverses at long stimulus-onset asynchronies (SOAs), giving rise to *inhibition of return*

Instead of presenting an arrow pointed to the possible target location, what happens if we just present a rapidly flashed stimulus at the potential location of the target? In general, we find that a peripheral stimulus onset can also summon attention to a location, similar to an arrow pointing to a specific location, but more rapidly and reflexively (Cheal & Lyon, 1991; Prinzmetal et al., 2005). Figure 11 shows idealized RT functions demonstrating the canonical effects of cueing attention using a central symbolic stimulus (i.e., like an arrow) versus with a peripheral stimulus that appears at the cued location (i.e., like a box appearing around the possible target location). These RTs are measured by varying the stimulus-onset asynchrony (or SOA) between the cue and the target across trials (Nakayama & Mackeben, 1989; Posner, 1980). The SOA is the time between when the cue first appears and when the target appears that subjects are required to report with a button press, saccade, or other behavioral response. The RT is measured as the time between when the target first appears and when the subjects make that behavioral response.

Several differences are notable. First, the functions relating RT to SOA for peripheral versus central cues have different time courses. A peripheral stimulus that suddenly appears at a location draws attention to that location much more quickly than does a central, symbolic cue. This is not entirely surprising. The central arrow is an arbitrary stimulus that needs to be interpreted (i.e., recognized and categorized) before the appropriate shift of attention can be programmed and executed (Müller et al., 1998). In contrast, the sudden onset of a stimulus in the visual field has strong bottom-up salience that appears to draw attention to itself more quickly, as described previously (Yantis & Jonides, 1984).

Besides resulting in faster deployments of attention, peripheral onsets appear to result in a subsequent effect that is not observed with central, symbolic cues. This effect is known as *inhibition of return* (Posner et al., 1985). Following a peripheral cue, we can see in Fig. 11 that subjects’ responses are initially faster when the target appears at that cued location, but after a period of time, typically about 300 ms in many experiments, the effect flips. That is, people take longer to respond to a target appearing at the cued location than to a target appearing at an uncued location (Abrams & Dobkin, 1994).

The term *inhibition of return* describes the hypothesis that has been proposed to explain this effect (Posner et al., 1985). The idea is that a subject initially shifts attention to the cued location, then after the target does not appear there within a few hundred milliseconds, they shift the spotlight of attention elsewhere in search of the target. This inhibits the cued location so that attention can be disengaged (or released) from that cued location. However, when the target then appears back at the cued location, the inhibition that freed attention from that location lingers and slows our processing of that target. The slowing of RT that is the inhibition of return effect has been extensively studied to understand the dynamics of attentional shifts in situations like the visual search tasks we discussed previously (Castel et al., 2003; Klein, 1988; McDonald et al., 1999; Snyder & Kingstone, 2000; Tipper et al., 1991).

In visual search, it has been proposed that inhibition of return works to facilitate attentional foraging, keeping attention moving to new locations in search of task-relevant information. However, it appears that inhibition of return may be due to activity in the oculomotor system related to planning saccadic eye movements with effects being absent when search is performed covertly without eye movements (as described in Mueller & von Muehlenen, 2000; Taylor & Klein, 2000). Thus, the origin of the inhibition of return effect is likely to be on the motor end of processing (Schall & Woodman, 2012), perhaps not being related to covert attentional selection by the sensory cortices of the brain.

The differences in the dynamics of attentional deployment following peripheral versus central cues has propelled the hypothesis that different kinds of visual attention were at work during these various types of cueing tasks (Prinzmetal et al., 2005). Specifically, researchers described cueing by central, symbolic cues as due to endogenous attention (i.e., from within), and cueing by peripheral cues as due to exogenous attention (i.e., from outside) (Coull et al., 2000; Theeuwes, 1991; Worden et al., 1995). These terms imply that different mechanisms of attention are at work when these different types of cues are used. However, recent studies of both neural activity (Gilbert & Li, 2013) and behavior (Awh et al., 2012) suggest that peripheral and central cues elicit shifts of the same visual attention mechanism, just with different temporal and spatial characteristics due to the differences in the stimuli used to cue locations. However, this remains a topic of debate in the empirical literature (Kasten et al., 2020; Keefe & Störmer, 2021).

## The locus of attentional selection

Up to this point, we have been discussing how attention is studied focusing mostly on behavioral measures and neuronal response properties. However, neuroscientific studies of attention have answered some of the most difficult questions about the nature of attentional selection (Corbetta & Shulman, 2002; Luck et al., 2000; Noudoost et al., 2010). One of these questions plagued attention researchers for decades during the twentieth century.

At what point in visual processing does attention select an input for preferential processing? The first theory of attention in the modern era was put forth by Donald Broadbent (1957). He proposed that attentional selection occurs early during the processing of sensory input to the brain. Broadbent’s theory became known as the *filter theory*. He proposed that attention selects a source of sensory input based on the presence of a low-level feature (e.g., the color red), before the object at that location has been recognized (i.e., determining that the red thing is a break light that requires quick action). Everything else that is not selected is filtered out prior to object recognition.

Filter theory makes the intriguing prediction that we will not know what the objects in our visual field are until we focus attention on them. As the study of attention unfolded, the findings of many experiments supported the predictions of filter theory, however, there were prominent falsifications of the prediction that unattended objects will not have been recognized (Treisman, 1969). This led to the counterproposal that we might recognize every object that appears in our visual field prior to attentional selection, with attention only determining which objects are then stored in memory (Deutsch & Deutsch, 1963). These opposing views lead to a stalemate of scientific opinions on the locus of attentional selection. This stalemate was broken by neuroscientific experiments that measured when in time, and where in the brain, attention effects are found.

If attention is selecting an input early during processing, then we should be able to measure it early in time. This is the beautifully simple logic that guided a series of influential studies of attention using electrophysiological recordings from human subjects (Hillyard et al., 1973; Picton et al., 1976). By recording subjects’ ERPs, researchers were able to determine that attention effects are observed at the earliest moments that electrical activity of the human brain is elicited by stimuli. Researchers had human subjects attend to a location in space to look for a certain direction of motion, then the researchers instructed the subjects to attend to different locations across different blocks of trials, presenting stimuli at both locations across trials regardless of where attention was directed (Hillyard et al., 1973). This design allowed scientists to compare the response of the brain to identical stimuli when they were attended versus ignored.

What is evident from these experiments is that attention boosts the amplitude of the potentials elicited by the stimuli from the earliest deflections onward. For example, the visual P1 component, elicited 70–90 ms post stimulus onset, shows reliable spatial attention effects (Anllo-Vento & Hillyard, 1996; Hillyard et al., 1973; Luck et al., 1994b; Mangun & Hillyard, 1990). This observation is consistent with the idea that attention works like a spotlight making a stimulus appear more clearly or brighter since these early components are larger when a stimulus is brighter (i.e., higher contrast with the background). However, the most critical aspect of this effect for the locus of selection debate is when it was measured. This increased response due to attention was evident within 70–90 ms after the visual stimulus was presented. This is a fast effect given that we know action potentials only start arriving at area V1 in the visual system about 40 ms after a stimulus onsets (Schmolesky et al., 1998). However, we do not know the anatomic origin of most human event-related potentials (Woodman & Schroeder, 2012), so it is not possible to directly relate the timing of this effect to a particular anatomical region of the brain (Woodman, 2010).

Recordings from macaque monkeys have provided even more support for the proposal that attention can select inputs early in the stream of processing (Pouget et al., 2012). Multiple groups of researchers used tasks like the cueing paradigms described above, in which a participant attends to a particular location in space, awaiting a target stimulus (Luck et al., 1997a; Maunsell, 1995; Moran & Desimone, 1985; Motter, 1993). This work has shown that attention effects are observed across all visual areas of the cortex, including area V1. Motter (1993) was the first to show that even neurons in V1 show elevated firing rates when attention is focused on an object in the neuron’s receptive field by placing attended items in cluttered arrays of to-be-ignored items. Others then found attention effects on the firing rates of V1 neurons in curve tracing and other complex attention tasks (Roelfsema et al., 1998; Supér & Roelfsema, 2005). Subsequent imaging research with human subjects indicates that attention effects may be measured as early as the lateral geniculate nucleus (or LGN) of the thalamus (Gandhi et al., 1999; Schneider & Kastner, 2009), which is the first anatomic structure that information is sent after leaving the retina and splitting into hemifield representations.

There is an alternative view that attentional selection does not always operate early in the brain (Treisman, 1969). Instead, the proposal states that where attention effects play out in the brain depends on the task at hand, with a locus of selection that can vary depending on the nature of the target (Lavie, 1995; Lavie & Tsal, 1994) and the number of inputs that are competing for attention (Boehler et al., 2009; Hochstein & Ahissar, 2002; Hopf et al., 2010; Lavie, 2005; Vogel et al., 2005b). If we search for a certain face in a crowd of people, then the activity that will be critical for this search will be in the visual areas with face selective cells (i.e., the superior temporal sulcus in macaques, or the fusiform face areas on humans) (Gross et al., 1972; Kanwisher et al., 1997; Wojciulik et al., 1998). The second critical factor is the density of input in the visual field (Cohen & Ivry, 1991), as we discussed previously in terms of the set size or number of objects in the visual field. If our task requires us to determine whether an object appears in a blank, homogeneous field (e.g., a plane appearing in sky on a cloudless day), then neurons in the visual system with huge receptive fields provide signals sufficient to detect these stimuli (Funahashi et al., 1989; Suzuki & Azuma, 1983). However, if our visual systems are being bombarded with visual inputs, then we may need to focus the receptive fields of neurons in the visual system on specific objects that appear target-like, filtering out the ignored inputs (Reynolds & Chelazzi, 2004), so that the responses of individual neurons can be useful (Luck et al., 1997b).

## Saliency maps and sources of visual attention

Previously I discussed the idea that both information about the salience of an object’s features (i.e., bottom-up factors) and information about the task relevance of an object’s features (i.e., top-down factors), jointly determine where attention is focused when we look out on the world (Awh et al., 2003; Kinchla & Wolfe, 1979b; Shulman et al., 1997). Where does this integration of visual information occur? Through this discussion of visual attention, we have described effects of visual attention in which an object or location becomes the focus of attention. But what does the focusing? This question essentially asks where the source or sources of attentional control are located in the visual system (Baluch & Itti, 2011; Gilbert & Li, 2013; Serences & Yantis, 2006). Surprisingly, there appears to be more than one part of the primate visual system that integrates different types of information and focuses attention.

**Fig. 12:**
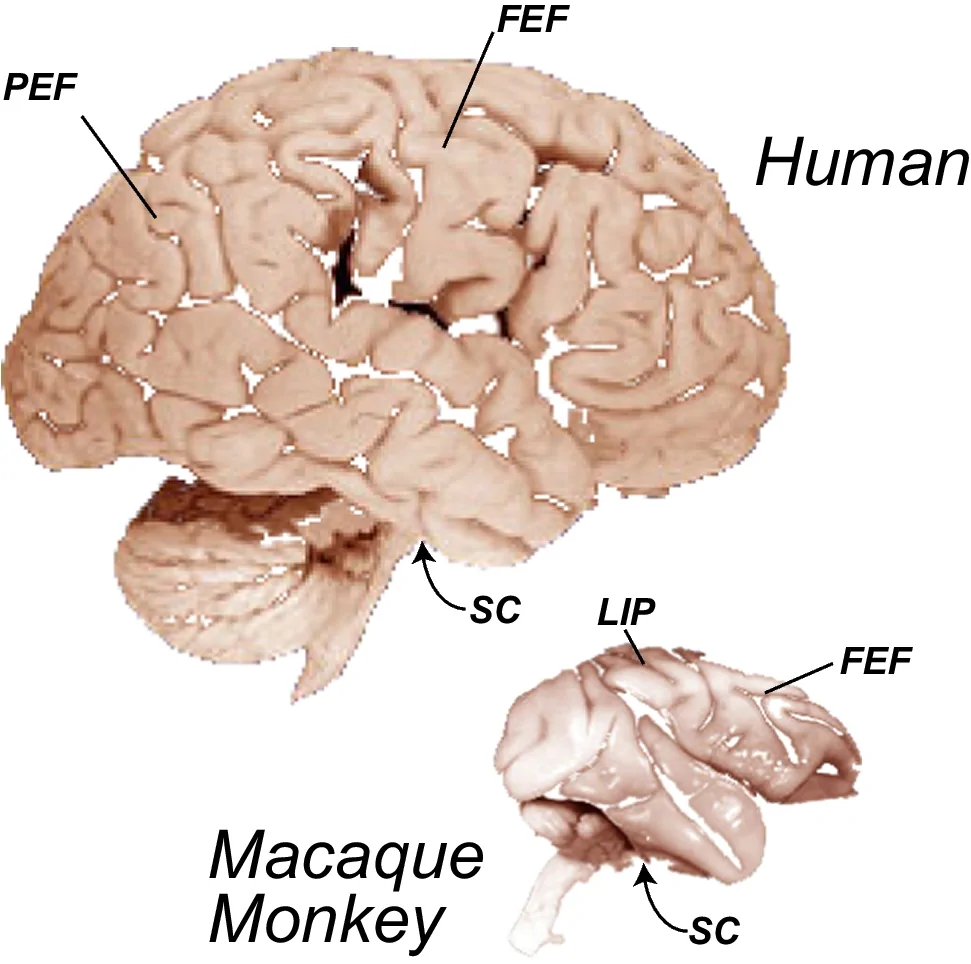
The regions of the human (top) and monkey brain (bottom) that integrate bottom-up and top-down attentional signals. The frontal-eye field (FEF), the lateral intraparietal area (LIP), and the superior colliculus (SC) appear to work as a circuit to control where attention is deployed in the visual field

One region of visual cortex that appears to be critical for controlling visual attention is known as the *frontal-eye field* (Noudoost et al., 2010; Schall, 2004). Figure 12 shows that the frontal-eye field is a region of prefrontal cortex, just anterior (i.e., in front of) supplementary motor areas in brains of humans and macaque monkeys. Inputs from across the entire visual cortex converge upon the frontal-eye field, making it ideally suited to integrate information derived from neurons across the visual system (Schall et al., 1995).

The firing rates of visually responsive neurons in the frontal-eye field do not exhibit the feature-specific tuning functions that dominate lower-level areas of visual cortex (Bichot et al., 1996; Thompson et al., 1996). Instead, these neurons fire at high rates for visual inputs that are jointly determined by the strength of bottom-up input from virtually any stimulus presented in the neuron’s receptive field and whether that object needs to be attended because it is behavioral relevant (Bichot et al., 2001; Thompson et al., 1997). For example, when a monkey is performing a visual search task and a target appears in the neuron’s receptive field, that neuron will fire action potentials at a higher rate than when a distractor appears in the receptive field (Schall & Hanes, 1993; Westerberg et al., 2023).

This evidence from neural recordings and similar findings from human imaging experiments has resulted in the view that the frontal-eye field represents a saliency map that controls where attention is shifted in the visual field (Hamker, 2004; Noudoost et al., 2010; Schafer & Moore, 2011). This idea is consistent with theories of visual attention that propose a map of locations integrates bottom-up and top-down influences into a spatial map that is used to control shifts of attention (Itti & Koch, 2000; Navalpakkam & Itti, 2005; Wolfe & Horowitz, 2004; Wolfe, 2020, 2021).

Can you control attention by controlling this area? Yes. If you inject microstimulation into the fontal-eye field, you can cause attention to be shifted to locations (Moore & Fallah, 2001). Moreover, the effects of that shift can be measured in posterior regions of the visual system, such as area V4 (Armstrong & Moore, 2007). Thus, the frontal-eye field has all the characteristics of the region of the brain that is in control of visual attention.

One answer to our question of what controls visual attention is the frontal-eye fields. However, our story is more interesting than that. The frontal-eye field is densely interconnected with two other areas that appear to perform much the same function of determining which visual inputs receive the benefit of attention (Schall & Thompson, 1999). One area is in the parietal lobe, known as the *lateral intraparietal* area (or *LIP* in Fig. 12). The other region is the subcortical *superior colliculus* (or *SC*). Figure 12 shows the relationship between these areas in the human and macaque brains. The properties of the neurons in these areas appear to be very similar to those of the frontal-eye field in controlling where attention is focused in the visual field (Buschman & Miller, 2007; Schmolesky et al., 1998). What is unclear is why visual attention has so many bosses. It is possible that each area controls a slightly different aspect of attentional selection. For example, posterior regions might perform bottom-up selection and frontal regions perform top-down selection (Buschman & Miller, 2007). It is also possible that evolution favors redundancy to allow for survival after brain injury or compensate for fluctuations in any given control structure. This will be an important question for the next generation of visual attention researchers to answer.

## Dividing attention: Attentional blinks and psychological refractory periods

Illusions can demonstrate important limitations in how our brains can interpret information from the world (Cretenoud et al., 2021). One of the most practically important illusions of our visual system is that we can pay attention to multiple things at the same time (Srna et al., 2018). Not long after researchers started talking about a spotlight of attention, they began wondering if it would be possible to split that spotlight and simultaneously attend to two locations at the same time (Shulman et al., 1979, 1985). Using a variety of tasks, the answer has often been that attention is not divisible except in split-brain patients (Luck et al., 1989, 1994a; McCormick et al., 1998). However, this debate continues (Gabbay et al., 2019; Golomb, 2019). I will begin by discussing the type of situation in which evidence favors the hypothesis that participants are attending to multiple objects or locations.

Can our visual system deploy attention to more than one spatial location at the same time? Studies using behavior, electrophysiology, and neuroimaging evidence appear to provide support for this idea (Awh & Pashler, 2000; Castiello & Umilta, 1992; Kramer & Hahn, 1995; McManis & Somers, 2004). However, all these methods are prone to at least two alternative explanations. First, averaging behavioral or neuroscientific data across trials means that a probabilistic deployment of attention across trials could look like we were attending to two things at the same time. For example, if we shift attention to the left on some number trials and to the right on other trials, then averaging across these trials would make it look like we focused 50% of our attention on each location (Woodman & Luck, 2003b). The other alternative explanation that vision scientists have discussed is that rapid shifts of a single focus of attention could also look like a split spotlight of attention.

Models of attention that propose attention selects multiple objects or locations at the same time also have stages of processing in which single items are handled individually, such as comparing items to the target held in memory (e.g., Bundesen et al., 2005). Consistent with this aspect of computational models, I will next discuss how our visual systems have great trouble when trying to handle more than one object during a variety of tasks.

**Fig. 13:**
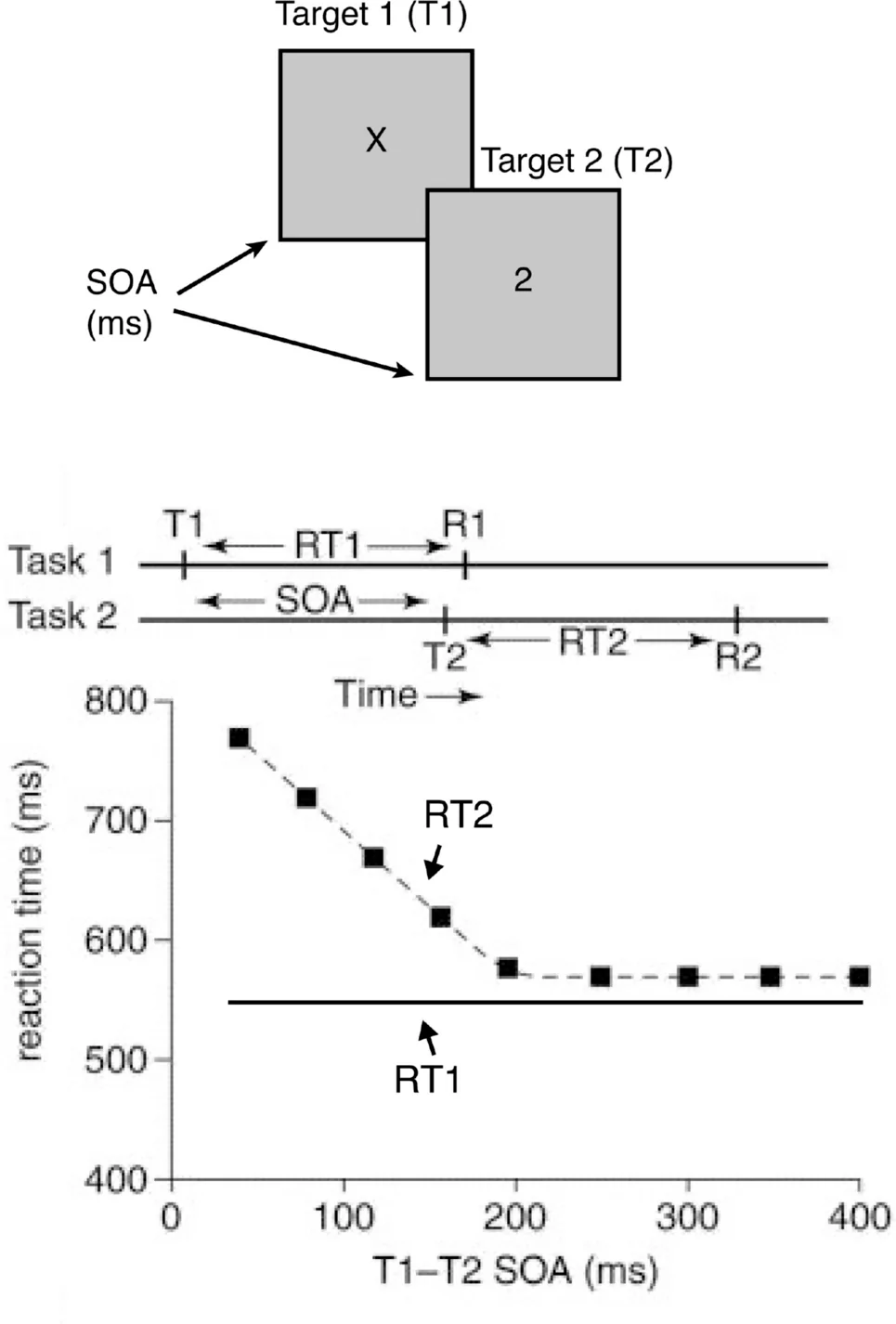
The Psychological Refractory Period Paradigm (or PRP) measures how fast observers can respond to two different simple stimuli (T1 and T2, see top panel). When those two visual targets appear close together in time (i.e., at a short stimulus-onset asynchrony, or SOA) then observers reaction time is delayed to the second target. Reprinted from Luck et al. (2000) with permission of Elsevier

Many researchers have looked at how attention operates when multiple objects need to be processed quickly at the same spatial location (Broadbent & Broadbent, 1987; Martens & Wyble, 2010; Telford, 1931; Welford, 1952; Zylberberg et al., 2012). We might imagine that this should be trivial since the spotlight of visual attention does not need to move to allow this to happen. Figure 13 shows an example of one of these experimental situations. In this situation, subjects are asked to respond to each of two targets as quickly as possible. In our example, the first target is either an X or an O, while the second target is either a 1 or a 2. The subjects need to press buttons as fast as possible to report the objects as they appear. Then, the researchers vary the time between the two targets (i.e., the SOA). For almost exactly a century now, researchers have found that when the time between the two objects is short (i.e., < 200 ms), the response to the second object is slowed relative to when it is responded to alone (Telford, 1931). However, when the time separating the two stimuli is longer (i.e., 300 ms), the response to the second object is similar to when the first target is present long before the second target, or when the first target is not presented at all.

This effect is known as the *psychological refractory period*, referring to the idea that the brain is exhibiting a refractory period similar to an individual neuron (Telford, 1931). During the neuronal refractory period, a neuron cannot fire another action potential because the sodium–potassium pump is reestablishing the resting membrane potential (Hausser, 2000; Hodgkin & Huxley, 1939, 1952). The analogy is that during the psychological refractory period, our minds cannot process another object (Pashler, 1994b). Research using behavioral and electrophysiological measures has revealed that the source of this psychological refractory period appears to be after perceptual processing has occurred (Luck, 1998; Pashler, 1994a, b). Given that the visual search data we saw in Fig. 3 suggests that attention shifted between two objects every 50 ms, it is perhaps not surprising that the locus of the interference in this psychological refractory period is not visual attention, but instead related to trying to select two responses at the same time (Pashler, 1994b). In summary, we cannot respond to two objects at the same time, and this has important real-world implications. But what happens in a similar situation in which subjects only need to remember two rapidly present stimuli and are not required to respond to each object as quickly as possible?

**Fig. 14:**
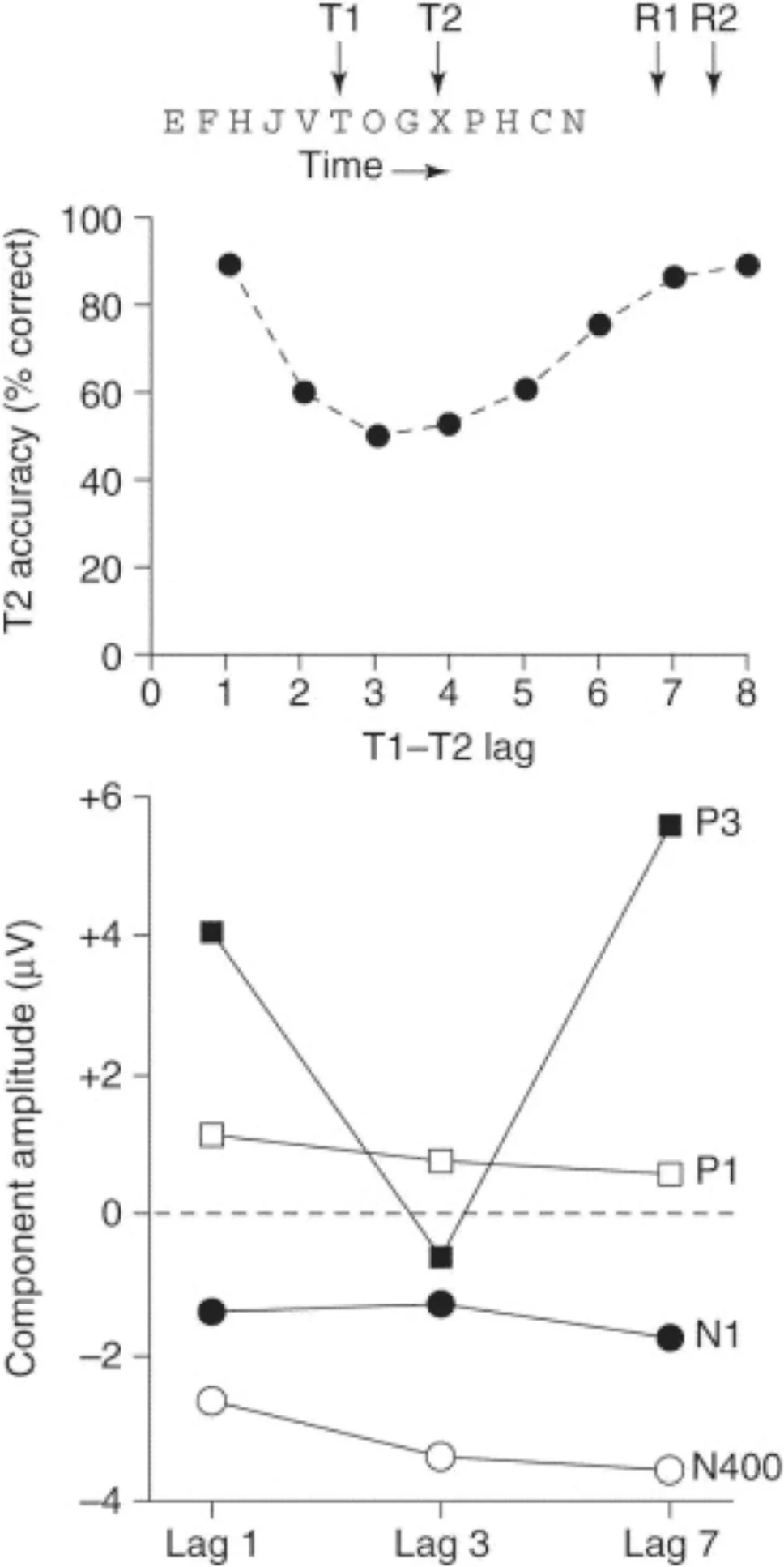
The Attentional Blink Paradigm and the results from event-related potential experiments that measured when during visual processing the brain’s response shows the attentional blink pattern. The top panel shows that subjects need to search for two target letters in the stream of visual stimuli and report whether they were present at the end of each trial. The middle panel shows the attentional blink effect in which people are impaired at reporting the second target (T2) when it closely follows the first target (T1). Lag 1 means the very next stimulus, lag 2 means two stimuli later, et cetera. The bottom panel shows the results from a series of experiments that measured the earliest visual ERPs (the P1 and N1) elicited by the T2 stimuli, the N400 related to recognizing the meaning of the stimulus, and the P3 related to encoding a stimulus into working memory. Only the late P3 showed the attentional blink effect, indicating that this effect is due to competition operating at a late stage of processing (i.e., visual working memory encoding). Reprinted from Luck et al. (2000) with permission of Elsevier

If you view a rapid serial visual presentation stream (RSVP stream, see Fig. 14), you can easily determine if a single target object or scene is present in the stream (Potter, 1975). For example, if I tell you to look for the letter T in a stream of letters and report whether it was present at the end of the trial, then this task is trivial for you (Shapiro et al., 1997). Performance is nearly perfect even when the stimuli are presented for just 10 ms each, with just 90 ms between each stimulus (Raymond et al., 1992). Now let us make your task slightly harder. I want you to report whether you see the letter T *and* the letter X in the stream. Again, you do not respond to these targets as they are presented, instead you make unspeeded responses at the end of each trial of the task. You simply need to remember them so that you can report their identity at the end of the stream. What is observed in these experiments is that your ability to report whether the second target was present is significantly impaired when it appears 200–600 ms after the first target (Luck et al., 1996; Martens & Wyble, 2010). Frequently in these experiments this impairment is measured in terms of the lag between the first and second target. As in Fig. 14, lag 2 means that the second target was presented two stimuli after the first. When stimuli are presented every 100 ms this makes lag 1 equal to 100 ms, lag 2 equal to 200 ms, and so forth.

This effect is known as the *attentional blink* (Raymond et al., 1992). The name refers to the original idea that this impairment to report the second target is because attention is focused on the first target immediately after it is presented and unavailable (i.e., the lid is closed) for the second stimulus. The result is an attentional effect that is like a physical blink of the eyes. During the attentional blink, our brain’s sensitivity to the new information in the second target is impaired or blocked analogous to how we cannot receive any new visual information from the world when we move our eyelids across our eyes to blink (Palmer, 1999). As attention dwells on this first target, it is not available to process another target object (Chun & Potter, 1995). However, note that people are not actually blinking, so that the information is getting into the brain, available for processing. Instead, it appears that processing is focused on the first target and the second target is mishandled by the visual system.

So, where during the stream of visual processing does the attentional blink occur? When we discussed the psychological refractory period, we discussed that the locus of that effect was extremely late, localized in time to the period when subjects are selecting which motor response to make to each stimulus (Pashler, 1994b). In the case of the attentional blink, it appears that perceptual processing of the stimuli may occur fairly normally with representations simply not being encoded into a durable form in working memory (Chun & Potter, 1995).

We can infer that perception of targets during the attentional blink can be performed by the brain from a series of experiments in which ERPs were recorded from subjects (Luck et al., 2000). These ERPs have excellent temporal resolution, allowing researchers to measure precisely when attentional blink effects emerge (Luck & Kappenman, 2012; Woodman, 2010). The sequence of ERP waveforms that we measure from people are named by their polarity, with a P for positive, N for negative, and a number indicating the ordinal position of the component or its timing (i.e., P3 for the third positive going component, or N400, for a negative going component with a 400-ms latency). A series of experiments showed that the ERPs elicited by the second targets during the attentional blink were completely normal until processing of these targets reached the waveforms related to storing that target in working memory (Luck et al., 1996; Vogel et al., 1998). Figure 14 shows that the amplitude of the electrical activity elicited by the second target was similar for the early P1, N1, and N400, whether these items were presented before, during, or after the attentional blink. Surprisingly, this included waveforms related to semantic analysis of the visual stimuli, that is, the N400 (Vogel et al., 1998). These measurements of brain activity showed that during the attentional blink, when we cannot report whether an object was present, our brains appear to recognize the objects and process how they are related to the other objects that we have recently seen.

It appears that during the attentional blink our brains can perceive and recognize the objects that are presented to us. The limitation is that we cannot encode these two target representations into working memory at the same time (Luck & Vogel, 2001; Luck et al., 1996). After encoding of a target object has begun, there appears to be a period before the system is ready to handle another target (Chun & Potter, 1995; Martens & Wyble, 2010). This was also shown with the measurements of ERPs (Luck et al., 1996; Vogel et al., 1998). Figure 14 shows that the P3 was reduced in amplitude during the attentional blink, indicating that the normal processing of the second target by subsequent late-stage processing mechanisms (i.e., decision making, response selection, and execution) was not possible during this period (Luck & Vogel, 2001).

A revolving door metaphor can be useful to think about this phenomenon. If one person gets in a revolving door and starts pushing, then there is a short period before the next slot can be entered so the next person can start the processes of gaining access to the building. It appears that gaining access to post-perceptual mechanisms has a similar structural limitation (Dux & Marois, 2009). Thus, the attentional blink may be due to an inability to encode a second object into working memory and awareness while the first object is accessing this entrance to the brain, although the field continues to consider alternative explanations (Amir et al., 2022; Martens & Wyble, 2010; Su et al., 2015).

Human subjects show dramatic interference when trying to respond to different stimuli at the same time in the psychological refractory period. This occurs even for simple objects (like the letter X and number 2) that require just the twitch of a finger to respond (Pashler, 1994b). During most tasks we perform outside the laboratory, the stimuli and responses are much more complex. Driving is an excellent example in which we need to attend to possible road hazards, recognize them, and respond in one of multiple ways (e.g., swerve, break, accelerate). This can be scary to consider with the increasing distractions provided by modern technology. Our smart phones and the navigation or entertainment systems in our cars themselves can be difficult to leave alone while driving a car, even though our survival depends on our performance.

**Fig. 15:**
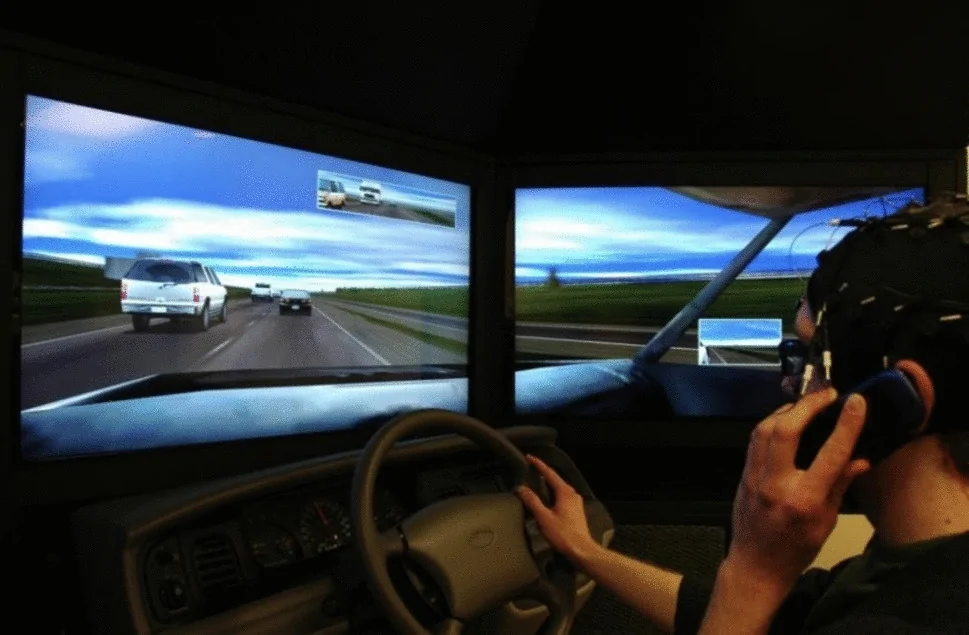
A participant in a driving simulator speaking with an experimenter on the phone. Image courtesy of David Strayer Ph.D., University of Utah

You might believe that the long time you have spent driving has made you an excellent multi-tasker capable of driving and doing something else (Watson & Strayer, 2010). However, the time spent driving does not appear to provide much aid in preventing dual-task interference (i.e., driving and talking on the cell phone at the same time) (Strayer et al., 2003). Cooper and Strayer (2008) used a driving simulator with subjects who reported a range of experience driving and talking on their cell phone (as shown in Fig. 15). In addition, they had all subjects practice talking on their cell phones and driving across many days. They found that neither the real-world practice nor the in-simulator practice reduced measures of interference. People were about 20% faster to hit the brake pedal in an emergency when not talking on the cell phone concurrently, and this interference was immune to practice.

As budding researchers, you might also say that driving requires visual processing and talking on the phone is a verbal task. As a result, these two tasks use different sensory systems and interference should be low (Strayer & Johnston, 2001). However, remember that when responses are required to be made to two stimuli, the interference plays out at the stage of response selection, not sensory input (Pashler, 1994b). Laboratory experiments have shown that the psychological refractory period effect is observed regardless of whether one stimulus is visual and one auditory, or one response made with the hand and one verbal (Pashler & Johnston, 1998). Spreading the tasks across input and output subsystems does not eliminate the interference.

Suppose that you decide to not respond to your phone conversation when driving becomes difficult? It appears that you still suffer from the type of interference measured in the attentional blink paradigm, even when stimuli are split across verbal and visual modalities (Arnell & Jolicoeur, 1999). This means that processing the information from the phone conversation could result in you missing an important visual target, like a red light. Wonder why we do not suffer from this interference when carrying on a conversation with someone in the car? Research shows that adult passengers in a car modulate their conversation rate as a function of driving demands, unlike a phone conversant that is unaware of the driver’s surroundings (Strayer & Johnston, 2001). That is, the other person in your car sees the traffic get heavy, the red light, or the truck bearing down on you, so they get quite to allow you to keep them alive.

## Continuing topics of debate

The previous sections of the review described the origin of the methods and the controversies regarding the study of visual attention. Several current topics of debate have grown out of these classic studies and findings. In the next section, I will review several of the questions regarding the nature of attentional selection that have engaged attention researchers in heavy debate during the present century of study. During the period from the publication year of 2000 to 2025, the number of papers with *attention* in the abstract or title increased by 300% (4,925 in 2000, 12,877 in 2025, PsycInfo, ProQuest, 2026). Thus, the current controversies from this period have also been studied at an intensity that is orders of magnitude more intense than in previous eras.

### Modulating attentional capture with value and learning

Several of the most intensely studied topics in visual attention have grown out of the attentional capture literature. One such topic is the phenomenon known as value driven priority (Anderson, 2013). If we have participants look for targets in visual search arrays, they can do this pretty quickly. However, Anderson and colleagues had participants perform a task in which they learn to associate responding to targets of a certain color with high reward (Anderson et al., 2011). When these subjects performed visual search for a target defined by its shape, the researchers found that distractor shapes presented in the color previously associated with high reward significantly slowed subjects’ responses to the actual target in the array. The idea is that visual features associated with high reward value outcompete other features for the benefit of attention. Theoretical accounts of these effects are complex as they include reward, cognitive control, and attentional circuitry in the brain (Anderson, 2019; Anderson et al., 2021; Bachman et al., 2020; Raymond & O'Brien, 2009).

During attentional capture tasks in which participants look for target objects, salient nontargets are presented, which slow responses to the actual target object (Remington et al., 1992; Theeuwes, 1991). What researchers later noticed was that when these critical, salient distractors were presented at a given location, targets were particularly slow to find targets at that location on the next trial (Wang & Theeuwes, 2018). The idea was that this shows some kind of learned distractor salience, such that the visual system automatically learns to guide attention to likely target locations. Consistent with this, if distractors are consistently placed at the same location across trials, then we can see evidence from RTs and eye movements that people increasingly avoid processing the objects that appear at these locations (Drennan & Gaspelin, 2026). Findings such as these have results in theories of attentional capture which propose an active inhibition or suppression mechanism that is tuned to inhibit distractor features within a trial (Sawaki & Luck, 2014; Wang & Theeuwes, 2018) and across trials (Anderson et al., 2021; Gaspelin & Luck, 2018).

The next current topic of debate to grow out of the attentional capture literature focuses on the source of top-down control of attention. Recall that evidence for contingent attentional capture comes from observations that items that have features matching the searched-for target will be most effective in capturing attention (Folk et al., 1992, 1993). Theories from this era accounted for such findings by proposing that holding a representation of an object or feature (like red) active in visual working memory was sufficient to mobilize attentional selection mechanisms to favor new inputs that matched that memory (Bundesen et al., 2005; Desimone & Duncan, 1995). However, in the last couple of decades, the field has found results of empirical studies complicating our story (Carlisle & Woodman, in press).

### Where are the memories that control attention?

One set of empirical findings that have shown that working memory is not the only source of top-down control are the learning effects described above in which people learn to avoid distractors (Awh et al., 2012; Chun & Jiang, 1998; Gaspelin & Luck, 2018; Theeuwes, 2025). Another type of evidence that has been used to argue for cognitive control of attention beyond a simple memory-input matching process, are findings that participants appear to have cognitive control over whether they direct attention toward or away from active memory representations. For example, people appear to be able to avoid memory-matching inputs if they know these will never match the targets they are looking for (Arita et al., 2012). Some have proposed that these items actually capture attention initially and then can be actively avoided afterward during visual search (Moher & Egeth, 2012). These empirical findings show that an active memory representation need not be only used to select items for preferential processing, but we can use this memory along with control settings provided by task rules to filter out irrelevant inputs.

There is another set of empirical observations that have challenged the idea that attention is always simply guided by an active working memory is that participants appear to pick between high-level attentional deployment strategies and even shift how they use different features to select items as the environment changes (Irons & Leber, 2018; Zhang & Leber, 2024; Zhang et al., 2024). In sum, several different types of empirical observations from visual attention experiments have shown that human observers have flexibility in how they use the contents of memory, although theoretical debates continue regarding the nature of this flexibility (Carlisle & Woodman, in press).

### Do real-world scenes and objects break our models?

How do the laboratory findings discussed in much of this paper generalize to the real-world search tasks that we started this paper looking at (i.e., looking for the boy in the yellow shirt)? This has been an important topic of study during the last several decades of attention research. Researchers using the internet’s nearly infinite catalog of photographs have made progress in understanding distractor suppression (Evans et al., 2011; Seidl et al., 2012), visual memory storage (Brady et al., 2008; Konkle et al., 2010), as well as perceptual expertise (Folstein et al., 2017; Gauthier et al., 2000; Wing et al., 2022). Our ability to render and control artificial scenes have allowed researchers to begin answering this question without surrendering the ability to manipulate the stimuli themselves (Hollingworth, 2004; Hollingworth et al., 2001). Methods have also evolved to include collecting responses from participants in the form of drawings, creating a visual-motor form of free recall (Bainbridge et al., 2019).

### The relationship between attention and awareness

The relationship between awareness and the term *attention* has received increasing scrutiny in the twenty-first century by researchers. The most common usage of the word attention outside of cognitive psychology is to mean applying the mind to a stimulus or event to be aware or consciousness of its content. The visual masking literature was introduced to four-dot masking (Enns & Di Lollo, 1997, 2000), in which a target in a visual search array can be easily masked by four small dots that linger around the location of the target after it briefly appears. This type of masking appears to interact with attentional selection mechanisms, where researchers believe that shifting attention to the target can result in attention arriving at a location that only has four dots visible. The four dots then overwrite the target object representation before you can be aware of it (Woodman & Luck, 2003a). These findings contributed to a larger view that the visual system and brain in general work through re-iterative processing, with attention and awareness being a result of a sufficient number of full processing cycles through the visual stream (Lamme & Roelfsema, 2000).

The focus of attention researchers on the nature of awareness has invaded subfields of research as diverse as binocular rivalry and mind wandering. In the study of binocular rivalry, researchers present competing visual inputs to each eye (Blake, 1989; Li et al., 2017). What people experience is that their perception of the stimuli is not a blend of what each eye is seeing but instead alternates between the two inputs with participants experiencing periods of seeing one input for up several seconds between seeing the situation reserve and they now become aware of the other eye’s input (Blake, 1989). Recent decades have seen vision researchers focused on rivalry ask how attentional selection is involved in determining which stimulus we are aware of at any given moment (Li et al., 2017; Mitchell et al., 2004). For example, binocular rivalry stops when attention is diverted away from the visual stimuli, suggesting that visual-spatial attention to a location gates whether the rivalry competition plays out in the brain (Zhang et al., 2011). In the study of mind wandering, research has typically focused on ideas related to internal versus external loci (Schooler et al., 2011), topics more common in clinical science than in the cognitive study of attention. Recent decades have seen a burst of research on lapses of attention, a synonym for mind wandering, in which researchers attempt to quantify whether a human participant was on or off task on a given trial (Hasenkamp et al., 2012; Schooler et al., 2011). It is notable that some view these large-scale cognitive failures as being independent of the selective attention mechanisms discussed throughout this review (Varao-Sousa et al., 2018). This is a relatively unexplored portion of the theoretical space that should be a target for future researchers, as I discuss more next.

### Is attention a single mechanism or multiple mechanisms that do selection?

A final way that attention is studied, modeled, and used in the psychological literature is as a univariate construct. What do I mean by that? In many of the non-cognitive areas of psychology it is common to use the term attention to refer to how *on task* the individual is on that trial (Boehm et al., 2021). These models conceptualize attention as a number between 0 and 1 on each trial (Reynolds & Heeger, 2009), with most such models relating this gain construct to the idea of alertness or arousal (Coull, 1998; Pribram & McGuiness, 1975). Often in cognitive psychology the concept analogous to the univariate attention construct is studied under the term *mind wandering* or *lapses of attention* (Andrillon et al., 2021; Seli et al., 2016), as described above, referring to the notion that the mind drifts off task, or at least the critical aspect that keeps us engaged in our environmental context (Mooneyham & Schooler, 2013). A growing body of research is combining the methods used to study the granular selection and storage of bits of information that I discussed in this review to see what information people have access to with methods used to study mind wandering and our abilities to stay on task (deBettencourt et al., 2015; Shelat & Giesbrecht, 2026; Shelat et al., 2026).

## Future directions and outstanding questions

What are the dynamics of the large-scale neural circuitry involved in attentional selection? Here I described how many different areas of the brain work in concert to determine what inputs receive the benefit of attention. An important question moving forward is how these large-scale neural networks operate in real-time. For example, when attention is drawn to an object due to the strength of its bottom-up input, does the network operate differently than when attention is driven in a top-down manner to a task-relevant object? Increasing sophistication of our neuroscientific arsenal means that these questions are increasingly tractable (Buschman & Miller, 2007; Ekstrom et al., 2008; Katsuki & Constantinidis, 2012; Westerberg et al., 2023; Woodman et al., 2022).

Can we increase the capacity of attention through training? We discussed how capacity limits are what require attention to select certain information, because we cannot process it all. But what if we could just get rid of those capacity limits? This has been the dream of researchers for over a century (Thorndike & Woodworth, 1901a, b; Woodworth, 1938). Interest has intensified recently with technology companies developing online games with the goal of training attention. Some of this interest is spurred by published findings that training can improve how much information attention can select at one time (Green & Bavelier, 2003; Jaeggi et al., 2008).

The literature clearly shows that enough training on a task can virtually eliminate evidence of capacity limitations on that specific task (Logan, 1988; Schneider & Shiffrin, 1977; Shiffrin & Schneider, 1977). This is how researchers study automaticity, that is, the processing of task information that is so efficient as to not to show evidence of capacity limits, like a flattening of visual search slopes (Schneider & Shiffrin, 1977) or the elimination of set size effects in general (Logan & Stadler, 1991). These impressive feats of processing appear to be brought about by extensive learning and storage of the relevant information in long-term memory (Logan, 2002). However, when you become automatic at a specific task, it does not increase your capacity to process other information, even from seemingly similar tasks (Thorndike & Woodworth, 1901a).

Outside of the study of task automaticity, experiments have found it difficult to modify attentional capacities limits in a general way using training with standard tasks (Boot et al., 2013; Shipstead et al., 2012). An additional hope is that new technologies of neurostimulation will decrease the capacity limits that require attention to focus processing on a limited number of visual inputs (Reinhart & Woodman, 2015), but these techniques need further study and development before they can be used by the public. Thus, an important topic for future research will be whether we can reduce our reliance on attention mechanisms by reducing or even eliminating the capacity limits that make them necessary.

What are the computations that attentional mechanisms perform? We previously discussed how attention appears to serve to filter out distracting objects so that the features of attended objects can be accurately bound (Treisman & Gelade, 1980). However, there appear to be far more sophisticated computations that attention may be performing rather than simply accurately combining multiple independent responses (Bundesen & Habekost, 2008; Bundesen et al., 2005; Hamker, 2004; Lu & Dosher, 2023; Navalpakkam & Itti, 2005; O'Reilly & Munakata, 2000; Wolfe, 2021). For example, recent theories have proposed that attention may work by normalizing activity across large pools of neurons (Reynolds & Heeger, 2009). The question of what computations depend on attention will continue to be an important topic of study.

Finally, how is attention related to memory? Some models of working memory propose that the capacity limits of this memory store are a result of only being able to attend to a few memory representations (Awh et al., 1998; Camos et al., 2018; Cowan, 2016; Nee & Jonides, 2011). Indeed, visual working memory is defined by its capacity limits, just like the defining characteristic of attention. A continued topic of study is how attention and working memory are related in the brain (Cowan et al., 2024).

## Summary

As our tour of the topic of attention draws to a close, it is important to step back from the tasks, phenomena, and neural circuitry, to focus on the larger question. Why do we appear to have useful information about a small subset of the objects that our sensory transducers provide?

The overarching answer to this is that there is a competition for limited capacity mechanisms of the brain (Duncan, 1996). We discussed how the receptive fields of neurons in visual areas like V4 or MT can be viewed as a limited-capacity mechanism (Desimone & Duncan, 1995). If the receptive field is asked to handle multiple objects at the same time, the system breaks down because the cell cannot deliver output about the nature of the objects that it is being stimulated by (Luck et al., 1997a, b). Thus, at the level of individual neurons in the brain, attention is needed to allocate that neuron to processing an attended object and filter out unattended one(s). This results in benefits for the attended object but also costs for those that go unattended. The selection by attention can occur quite early in the visual system, such as at the level of the LGN and V1 neurons. However, it also appears that when our visual fields does not contain many competitors, selection can operate much later in the stream of visual processing (Hochstein & Ahissar, 2002).

I next discussed how this competition continues if visual information needs to be responded to quickly or stored in memory. I showed how trying to respond to two stimuli at the same time results in its own competition for access to response selection mechanisms. This type of scare resource is critical for the performance of real-world tasks in which performing two things at the same time results in interference. Finally, the attentional blink showed us that a limited amount of information can be encoded into working memory (also known as short-term memory) at the same time.

This summary should illustrate a point that has been implicit in my discussion but has not been made explicit yet. That is, attention is not one thing (Woodman et al., 2001). There appears to be multiple attentional selection mechanisms that help focus our limited-capacity mechanisms on the visual inputs that are most relevant for our survival (Schall & Woodman, 2012). Some operate early to disambiguate the responses of neurons early in the visual system. Others provide top-down control so that we are sensitized to select the objects in our world that are most relevant for us. Still, other selection mechanisms gate access to working memory storage or response stages of processing. The fact that we use a singular noun, attention, to describe a collection of selection mechanisms is a misnomer that likely results in fewer people knowing what attention is than James had hoped (James, 1890).

## Data Availability

Not applicable.
